# Genome-wide mRNA profiling identifies X-box-binding protein 1 (XBP1) as an *IRE1* and *PUMA* repressor

**DOI:** 10.1007/s00018-021-03952-1

**Published:** 2021-10-12

**Authors:** Magdalena Gebert, Aleksandra Sobolewska, Sylwia Bartoszewska, Aleksandra Cabaj, David K. Crossman, Jarosław Króliczewski, Piotr Madanecki, Michał Dąbrowski, James F. Collawn, Rafal Bartoszewski

**Affiliations:** 1 Department of Biology and Pharmaceutical Botany, Medical University of Gdansk, Hallera 107, 80-416 Gdansk, Poland; 2 Department of Inorganic Chemistry, Medical University of Gdansk, Gdansk, Poland; 3 Laboratory of Bioinformatics, Nencki Institute of Experimental Biology of the Polish Academy of Sciences, Warsaw, Poland; 4 Department of Genetics, Heflin Center for Genomic Science, University of Alabama at Birmingham, Birmingham, AL 35233 USA; 5 Department of Cell, Developmental and Integrative Biology, University of Alabama at Birmingham, Birmingham, AL 35233 USA

**Keywords:** XBP1s, UPR, ER stress, *BBC3*, XBP1u, *ERN1*

## Abstract

Accumulation of misfolded proteins in ER activates the unfolded protein response (UPR), a multifunctional signaling pathway that is important for cell survival. The UPR is regulated by three ER transmembrane sensors, one of which is inositol-requiring protein 1 (IRE1). IRE1 activates a transcription factor, X-box-binding protein 1 (*XBP1)*, by removing a 26-base intron from *XBP1* mRNA that generates spliced *XBP1* mRNA (*XBP1s*). To search for XBP1 transcriptional targets, we utilized an *XBP1s*-inducible human cell line to limit XBP1 expression in a controlled manner. We also verified the identified XBP1-dependent genes with specific silencing of this transcription factor during pharmacological ER stress induction with both an N-linked glycosylation inhibitor (tunicamycin) and a non-competitive inhibitor of the sarco/endoplasmic reticulum Ca^2+^ ATPase (SERCA) (thapsigargin). We then compared those results to the *XBP1s*-induced cell line without pharmacological ER stress induction. Using next‐generation sequencing followed by bioinformatic analysis of XBP1-binding motifs, we defined an XBP1 regulatory network and identified XBP1 as a repressor of *PUMA* (a proapoptotic gene) and *IRE1* mRNA expression during the UPR. Our results indicate impairing IRE1 activity during ER stress conditions accelerates cell death in ER-stressed cells, whereas elevating XBP1 expression during ER stress using an inducible cell line correlated with a clear prosurvival effect and reduced PUMA protein expression. Although further studies will be required to test the underlying molecular mechanisms involved in the relationship between these genes with XBP1, these studies identify a novel repressive role of XBP1 during the UPR.

**Supplementary Information:**

The online version contains supplementary material available at 10.1007/s00018-021-03952-1.

## Introduction

Endoplasmic reticulum (ER) stress can disrupt the folding and maturation of the secretory and membrane proteins and lead to the accumulation of unfolded proteins in the ER lumen, interruption of lipid synthesis, and deregulation of cellular calcium levels [1, 2]. The buildup of misfolded proteins in ER leads to the activation of the unfolded protein response (UPR), a multifunctional signaling pathway that either promotes cell recovery [3], or initiates cell death if the ER stress remains unmitigated [4]. The UPR signaling pathways are initiated by three ER transmembrane sensors: inositol-requiring protein 1 (IRE1), protein kinase RNA-like ER kinase (PERK) and activating transcription factor 6 (ATF6). IRE1 removes a 26-base intron from X-box-binding protein 1 (*XBP1)* mRNA in an unconventional splicing reaction that results in a translational frameshift that leads to the production of a functional and highly active spliced XBP1 (XBP1) transcription factor [5–9]. XBP1 enhances the expression of ER-resident chaperones and genes involved in ER-associated protein degradation (ERAD) [10] and promotes ER expansion [9]. The ER protein load is reduced by PERK-mediated phosphorylation of eIF2α which inhibits most protein synthesis, by ER-associated degradation of misfolded proteins, and by IRE1-mediated mRNA cleavage and degradation [3]. However, if ER stress remains unmitigated, the UPR utilizes the same pathways to promote cell death by activating the intrinsic apoptotic pathways [11–16].

The interruption of the UPR transitions from prosurvival to apoptosis and the alteration of cell fate decisions contribute to the pathomechanisms of a number of human diseases including diabetes mellitus, cancer, and neurodegenerative and respiratory disorders [17]. To facilitate novel interventions for treating these disorders, it is important to understand the mechanisms governing the UPR pathways. Although we know the many of the details of the UPR pathways that contribute to cellular survival or apoptosis [18–20], it remains unclear how these signals determine the cell fate transitions in vivo. Understanding molecular mechanisms underlying UPR-related cell fate decisions is very challenging given that the experimental models rely on pharmacological ER stressors that are used at different concentrations and utilize divergent mechanisms for disturbing ER homeostasis [21–24]. Furthermore, previous studies have shown that UPR signaling has distinct consequences that ultimately depend on the nature and intensity of the stimulus as well as the specific cell type involved [18].

Although previous studies suggested that the transcriptional activity XBP1 is important in deciding cell fate in the UPR [25–30], the information regarding XBP1’s direct role in modulating the transition between survival to apoptosis is limited. Therefore, the studies presented here were designed to select for XBP1-specific transcriptional targets and their roles in cell fate decisions. In our approach, we used inducible human cell lines that allowed for comparable and controlled expression of spliced XBP1 and unspliced XBP1 proteins. We also verified the identified XBP1-dependent genes with specific silencing of this transcription factor during mild pharmacological ER stress induction with both an N-linked glycosylation inhibitor (tunicamycin, Tm) as well as a non-competitive inhibitor of the sarco/endoplasmic reticulum Ca^2+^ ATPase (SERCA) (thapsigargin, Tg). Using next‐generation sequencing (NGS) followed by bioinformatic analysis of XBP1-binding motifs, and validation using XBP1-specific silencing and quantitative real‐time PCR (qRT‐PCR), we defined an XBP1-dependent regulatory network and identified XBP1 as a repressor of both *PUMA* and *IRE1* expression during the UPR. This approach not only confirmed previously known XBP1 roles during UPR, but also resulted in the identification of novel targets of this transcription factor that could fine-tune cell fate decisions. Furthermore, we show that XBP1 can modulate the PERK pathway activity via modulation of both *CHOP* and growth arrest and DNA damage-inducible protein (*GADD34)* mRNA expression. Although further studies will be required to test the underlying molecular mechanisms involved in the relationship between these genes with XBP1, the studies presented here identify a novel regulatory role of XBP1 during the UPR.

## Materials and methods

### Cell lines and culture conditions

HeLa S3 cells were obtained from the American Type Culture Collection (CCL‐2.2; Manassas, VA, USA). Cells were cultured in Minimum Essential Modified Eagle’s Medium (Thermo Fisher Scientific, Waltham, MA, USA) with 2 mM *l*‐glutamine (MilliporeSigma, Burlington, MA, USA), antibiotics (100 U/ml of penicillin and 100 µg/ml of streptomycin (MilliporeSigma), and 10% fetal bovine serum in a humidified incubator at 37 °C in 5% CO_2_ in 6‐well plates. Cells were allowed to grow to 70–80% confluence before the start of the experiments. The culture conditions for the human Schwann cells (SNF96.2), human epidermal keratinocytes (HaCaT), 10 donor-pooled human umbilical vein endothelial cells (HUVEC), and human bronchial epithelial cells (16HBE14o-) were the same as previously described in [21, 31].

The inducible HeLa S3 *XBP1s* and *XBP1u* cell lines were constructed beginning with vectors containing the cDNA sequences of *XBP1s* (NM_001079539.1) and *XBP1u* (NM_005080.3) that were obtained from GeneCopoeia (Rockville, MD, US; *XBP1s* cat. no. EX-Z4299 and *XBP1u* cat. no. EX-F0758). The ORFs sequences were verified with Sanger sequencing, and *XBP1s* and *XBP1u* cDNAs were restricted with EcoRI/MluI and EcoRI/BamHI, respectively, and cloned into the pCW57-MCS1-P2A-MCS2 (Hygro) vector that permits doxycycline-controlled inducible lentiviral expression [32]. pCW57-MCS1-P2A-MCS2 (Hygro) was a gift from Adam Karpf (Addgene plasmid # 80922; http://n2t.net/addgene:80922). The correct pCW57-*XBP1s* and pCW57-*XBP1u* insert sequences were verified with Sanger sequencing. These vectors along with VSV-G envelope expressing plasmid (pMD2.G) and lentiviral packaging plasmid (psPAX2) were used to transfect HEK-293 cells (ATCC CRL-1573) to generate lentiviruses carrying the *XBP1s* or *XBP1u* transgenes. The pMD2.G and psPAX2 plasmids were a gift from Didier Trono (Addgene plasmid # 12259; http://n2t.net/addgene:12259 and Addgene plasmid # 12260; http://n2t.net/addgene:12260, respectively). The lentiviruses were also used to transduce Hela S3 cells [33, 34]. Finally, following hygromycin b selection (300 µg/ml, Sigma) and qPCR verification of 24 h doxycycline induction (400 µg/ml, D3072 MilliporeSigma) of *XBP1s* and *XBP1u* mRNAs in HeLa S3 cells, two stable clonal cell lines capable of stable inducible expression of *XBP1s* (Hela-*XBP1s*) and *XBP1u* (HeLa-*XBP1u*) were obtained. These cell lines were cultured in Minimum Essential Modified Eagle’s Medium with 2 mM *l*‐glutamine, hygromycin B (300 µg/ml) and 10% tetracycline free fetal bovine serum (Takara Bio, USA) in a humidified incubator at 37 °C in 5% CO_2_ in 6‐well plates. Cells were allowed to grow to 70–80% confluence before the start of the experiments.

### Induction of ER stress and activation of the UPR

Pharmacological induction of ER stress and activation of the UPR were performed as we previously described [21]. Briefly, cells were treated with the compounds for the time periods specified: tunicamycin (Tm 2.5 or 0.5 μg/ml; Sigma, T7765), thapsigargin (Tg 50 or 2.5 nM; Sigma, T9033). CTRL cells were treated with vehicle CTRL, DMSO (< 0.5% v/v; Sigma, D2650). Furthermore, to verify IRE1 activity, cells treated were 20 µM 4µ8C (an IRE1 inhibitor, Sigma-Aldrich, SML0949) dissolved in DMSO (Sigma-Aldrich, St. Louis, MI, USA) [35].

### Real-time cell viability assay

For real‐time monitoring of cell viability, we applied the xCELLigence system as we described previously [36]. Briefly, HeLa cells (12,000 cells per well) were seeded in the 16‐well PC plates (00300600890, ACEA Biosciences Inc., San Diego, CA, USA) 24 h prior to the experiment. CTRL cells were cultured in the presence of DMSO vehicle. Treated cells were incubated with ER stressors for the next 24 h, and every 15 min, the cell conductances (cell index) were recorded. All experiments were performed in triplicate with three independent repeats. RTCA software v. 1.2.1 (ACEA Biosciences, Inc, San Diego, CA, USA) was used to calculate the normalized cell index and the cell growth curve slopes.

### Monitoring caspase 3 and caspase 7 activity

The caspase 7 is considered to be redundant with caspase 3 because these enzymes share an optimal peptide recognition sequence and have several endogenous protein substrates in common [37]. While our main goal was to assess caspase 3 activity, the commercially available assays do not distinguish between these two cysteine proteases. Hence, we applied the caspase‐Glo 3/7 assay (Promega, Madison, WI, USA) to measure relative caspase activity as described previously [21, 36]. Briefly, cells the day after transfection with the specified siRNA were seeded onto 96-well luminescence assay white plates with clear bottoms (Corning Inc., 3903). The next day, the cells were treated with ER stressors or vehicle (0.1% DMSO) for indicated time points. Following treatment, cells were washed with PBS and the Caspase-Glo 3/7 assays (Promega) were performed in accordance with the manufacturer’s instructions using the GloMax-Multi + Detection System (Promega). The results were normalized to the values obtained from the vehicle control treatments.

### siRNA transfections

siRNAs against *XBP1* (Ambion assay id s14915) and *BBC3* (Ambion assay id s25840) were purchased from Ambion. HeLa cells were transfected using the Lipofectamine RNAiMax (Invitrogen 13778030) according to manufacturer’s protocol. The siRNAs were used at final concentrations of 30 nM. The transfected cells were cultured for 2 days prior to further analysis. Ambion siRNA Negative Control 1 (Ambion assay id MC22484) was used as a control.

### Isolation of RNA

Total RNA (containing both mRNA and miRNA) was isolated using miRNeasy kit (Qiagen). RNA concentrations were calculated based on the absorbance at 260 nm. RNA samples were stored at −70 °C until use.

### Next‐generation RNA sequencing analyses

The RNA isolation and analyses were performed in HeLa-*XBP1s* and -*XBP1u* cells. Briefly, following XBP1 induction with doxycycline (24 h and 400 µg/ml final concentration) total RNA isolation, samples were validated with qRT‐PCR for ER stress activation prior to further analysis. Following rRNA depletion, the remaining RNA fraction was used for library construction and subjected to 100-bp single-end sequencing on an Illumina HiSeq 2000 instrument (San Diego, CA, USA). Sequencing reads were aligned to the Gencode human reference genome assembly (GRCh38 p7 Release 25) using STAR [38]. Transcript assembly and estimation of the relative abundance and tests for differential expression were carried out with Cufflinks and Cuffdiff [39]. The resulting data were validated with qRT‐PCR. The heat map generation and hierarchical clustering were performed with the Morpheus Web server (https://software.broadinstitute.org/morpheus). The Enrichr Web server (https://amp.pharm.mssm.edu/Enrichr/) [40] was applied to assign the NGS results into the ‘Gene Ontology Biological Process’ categories with the selection based on a False Discovery Rate *Q*‐value *q* < 0.05. Furthermore, the analyses were limited to experimentally verified interactions and no extended gene enrichment set analyses were performed.

### Measurement of mRNA quantitative real-time PCR (qRT-PCR)

We used TaqMan One-Step RT-PCR Master Mix Reagents (Applied Biosystems) as described previously [41, 42] using the manufacturer’s protocol (retrotranscription: 15 min, 48 °C). For NGS data validation, 96 custom TaqMan expression array plates (id) were used according to the manufacturer’s instructions. The relative expressions were calculated using the 2^−ΔΔCt^ method [43] with the glyceraldehyde 3-phosphate dehydrogenase (*GAPDH*), and neutral ribosomal phosphoprotein P0 *(RPLP0)* genes as reference genes for the mRNA. TaqMan probes ids used are provided in Supplemental Table 1.

### Western blots

The XBP1 protein detection was performed as described in [44]. Briefly, cells were lysed on ice for 15 min in RIPA buffer [150 mM NaCl, 1% NP-40, 0.5% sodium deoxycholate, 0.1% SDS, and 50 mM Tris–HCl (pH 8.0)] supplemented with Protease Inhibitor Complete Mini (000000011836170001; Roche, Basel, Switzerland). The insoluble material was removed by centrifugation at 15,000*g* for 15 min. Protein concentrations were determined by Bio‐Rad Protein Assay [Bradford‐based method; Bio‐Rad, Hercules, CA, USA] using bovine serum albumin (BSA; MilliporeSigma) as the standard. Following the normalization of protein concentrations, lysates were mixed with an equal volume of 2 times Laemmli sample buffer (Bio-Rad) and incubated for 5 min at 95 °C before separation by SDS-PAGE on stain-free TGX gradient gels (Bio‐Rad). Following SDS-PAGE, the proteins were transferred to PVDF membranes (300 mA for 90 min at 4 °C). The membranes were then blocked with BSA dissolved in PBS and Tween-20 (3% BSA and 0.5% Tween-20) for 1–2 h followed by immunoblotting with the primary antibody for each experiment for spliced XBP1 (mAb 12782; diluted at 1:1000; Cell Signaling Technology, Danvers, MA, USA) or unspliced XBP1 (NBP1-77681; diluted at 1:700; Novus Biological USA). The unsliced XBP1 antibody has been independently validated with siRNA against *XBP1* (Supplemental Figure S1). For PUMA, the monoclonal antibody MBS9131466 (MyBioSource Inc. San Diego, CA USA) was used for overnight incubations at 1:1500 dilution. For IRE1 (phosphor-S724), the monoclonal antibody ab243665 (Abcam, USA) was used at incubations at 1:1000 dilution. After the washing steps, the membranes were incubated with goat anti‐rabbit IgG (H + L) horseradish peroxidase-conjugated secondary antibodies (Bio‐Rad) and detected using ECL (Amresco, Solon, OH, USA). Densitometry was performed using Image Lab software v.4.1 (Bio-Rad).

### XBP1 motif analysis

The promoters of the gene transcripts that were affected by *XBP1* induction in the NGS experiments were analyzed for XBP1-binding sites. In each gene promoter sequence that was defined as a 20 kb window around the TSS, we examined only the open chromatin regions that were established in the HeLa S3 cell line by the ENCODE [45] project. We merged both DNase I-seq HeLa datasets found in Ensembl (v.79) [46]. We used the Nencki Genomics Database (v. 79_1) [47] to obtain genomic coordinates of these motif instances. For each gene, we calculated the number of instances found in the open chromatin regions.

### Statistical analysis

Results were expressed as a mean ± standard deviation. Statistical significance was determined using the Student’s *t*-test and ANOVA on ranks with *P* values *P* ≤ 0.05 considered significant. The correlation was accessed via the Pearson product-moment correlation method.

## Results

Since our working hypothesis was that the commonly used concentrations of ER stressors lead to non-physiological elevation of UPR signals and the potential for misassignment of UPR pathways targets or their role in cell fate decisions, we compared commonly used classical pharmacological stressors at high concentrations (high stress) and at low concentrations (low stress). We tested a glycosylation inhibitor tunicamycin (Tm) that is normally used at 2.5 µg/ml and a non-competitive inhibitor of the sarco/endoplasmic reticulum Ca^2+^ ATPase thapsigargin (Tg) that is normally used at 50 nM (high stress). For the low-stress model, we used Tm at 0.5 µg/ml and Tg at 2.5 nM. These concentrations were determined experimentally as the lowest concentrations that were able to induce *XBP1*, *HSPA5* (*BiP*) and *DITT3* (also known as *CHOP*) mRNAs by at least twofold after 6 h of treatment. We used HeLa cells since this is a common model system that has been employed in ER stress and UPR studies [48–51].

**Fig. 1:**
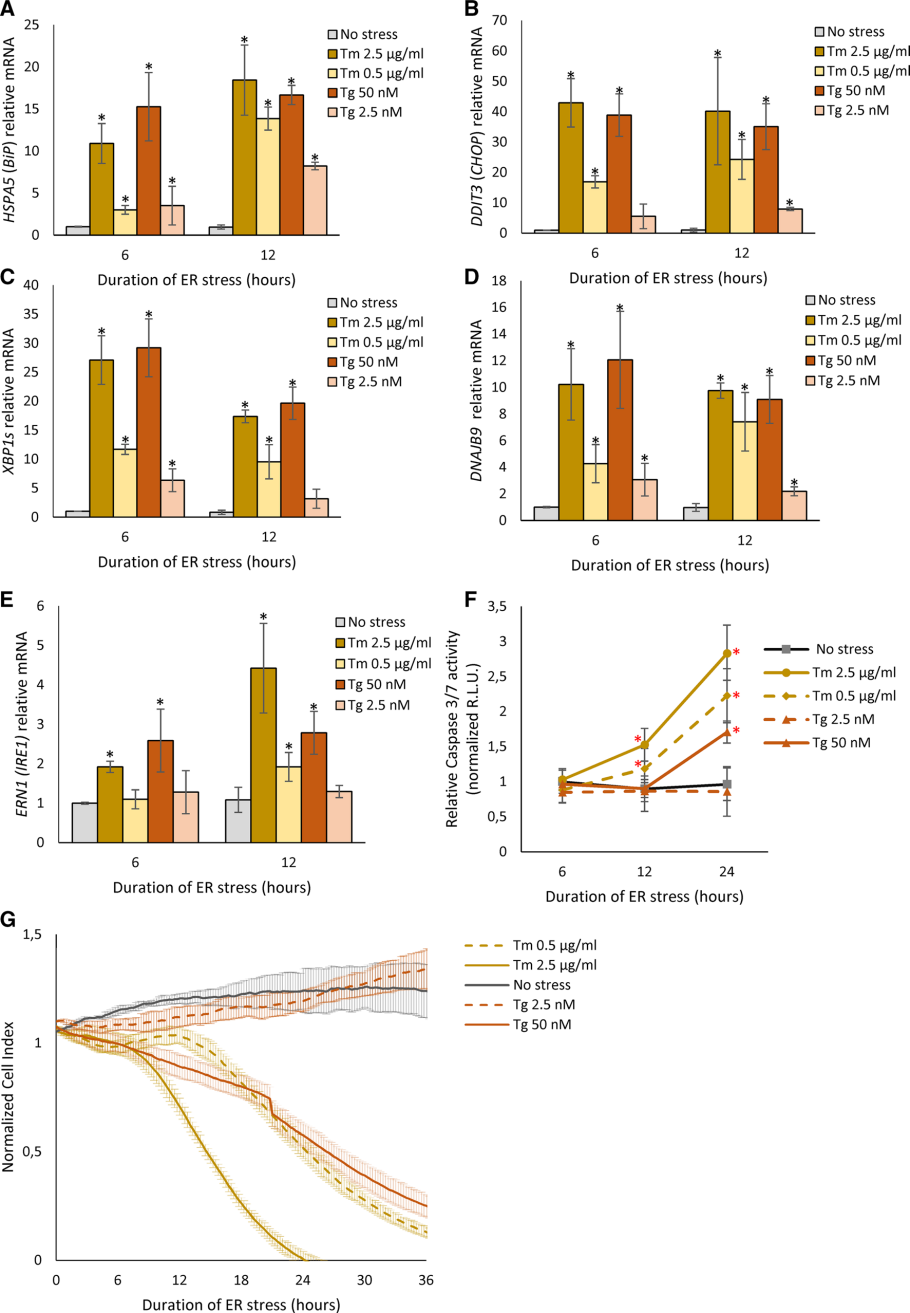
ER stress-induced changes in **A** *BIP*, **B** *CHOP*, **C** *XBP1s*, **D** *DNAJB9*, and **E** *IRE1* mRNA levels in HeLa cells. The results from three independent experiments (*n* = 9) are plotted normalized to *GAPDH* and *RPLP0* mRNA levels and expressed as a fold change over the no-stress controls. Error bars represent standard deviations. Significant changes (*P *value *P* < 0.05) are marked with an asterisk. ER stressors used: Tm (2.5 µg/ml), Tm (0.5 µg/ml), Tg (50 nM)) and Tg (2.5 nM). **F** HeLa cells were treated with ER stressors ((2.5 µg/ml), Tm (0.5 µg/ml), Tg (50 nM) and Tg (2.5 nM)) for time points specified. The caspase 3/7 activity was monitored by luminescence and expressed in relative light units (RLU). Cells for each time point were seeded in triplicate, and the experiments repeated three times. Error bars represent standard deviations. **G **The cell conductances (expressed as normalized cell index) were accessed every 15 min following a 36-h treatment with Tm (2.5 µg/ml), Tm (0.5 µg/ml), Tg (50 nM) and Tg (2.5 nM). DMSO was used in the no-stress conditions (CTRL). The conductances were normalized to the last value prior to experiment start. Representative results from three independent experiments measurements (*n* = 9) are plotted

As shown in Fig. 1, both stress models were able to induce both prosurvival (*HSPA5*, *XBP1s* and *DNAJB9)* and apoptotic (*DDIT3*) reporters. The adaptive BiP chaperone mRNA levels (*HSPA5*) was continuously elevated in both the high and low-stress models (Fig. 1A). High ER stress was effectively induced with the commonly used Tm and Tg concentrations (2.5 µg/ml and 50 nM, respectively) [21, 44, 49]. High stress resulted in a 10- and 15-fold induction of *HSPA5* mRNA with Tm and Tg, respectively, after 6 h and remained elevated after 12 h of treatment. In contrast, during mild ER stress conditions after 6 h, the *HSPA5* mRNA was induced by ~ threefold by both Tm and Tg, and this dramatically increased after 12 h of treatment (Fig. 1A). The proapoptotic *DDIT3 (CHOP)* mRNA levels were also elevated 18- and 6-fold with Tm and Tg treatment, respectively, after 6 h of low-stress conditions and remained elevated after 12 h (Fig. 1B). The use of the higher concentrations of Tm and Tg resulted in ~ 40- and ~ 35-fold inductions of *DDIT3 (CHOP)* mRNA expression after both 6 and 12 h. A similar pattern of high- and low-stress treatments was seen with *XPB1s* (Fig. 1C) and *DNAJB9* (Fig. 1D) mRNAs in that they were induced less during the low-stress conditions as might be expected. Interestingly, the *XBP1s* mRNA levels decreased after 12 h, whereas all the other mRNAs either increased or remained the same after high- and low-stress conditions. The *DNAJB9* (DnaJ heat shock protein family (Hsp40) member B9) mRNA levels, a pro-adaptive chaperone and an XBP1 transcriptional target, were elevated as expected under both high- and low-stress conditions (Fig. 1D)*.* Furthermore, *ERN1* (*IRE1*) mRNA expression was induced after 6 h only by higher Tm and Tg concentrations, whereas in mild stress, *ERN1* mRNA was only elevated by Tm treatment after 12 h (Fig. 1E).

We next tested the effects of high and low stress on cell viability and proliferation. As shown in Fig. 1F, the Tm treatments at high- and low-stress conditions elevated caspase 3/7 activity at 12 and 24 h, whereas the Tg treatment elevated the caspase activity only at 24 h during high stress conditions (Fig. 1F). The lack of significant apoptotic signal for the lower Tg concentration was consistent with the lower induction of apoptotic mRNAs. These observations were also consistent with the results of real-time monitoring of HeLa cell proliferation (Fig. 1G). The mild ER Tg-induced stress had no significant effect on HeLa proliferation up to 36 h, while treatment with higher Tg concentration significantly limited cell growth throughout the entire time course. Whereas, both Tm concentrations affected HeLa growth and demonstrated a clear indication of apoptosis after 18 h (Fig. 1G). Taken together, the data illustrate that lower stress conditions still activate the UPR and may provide a more physiological model to delineate the differences between the adaptive and apoptotic UPR signaling pathways.

**Fig. 2:**
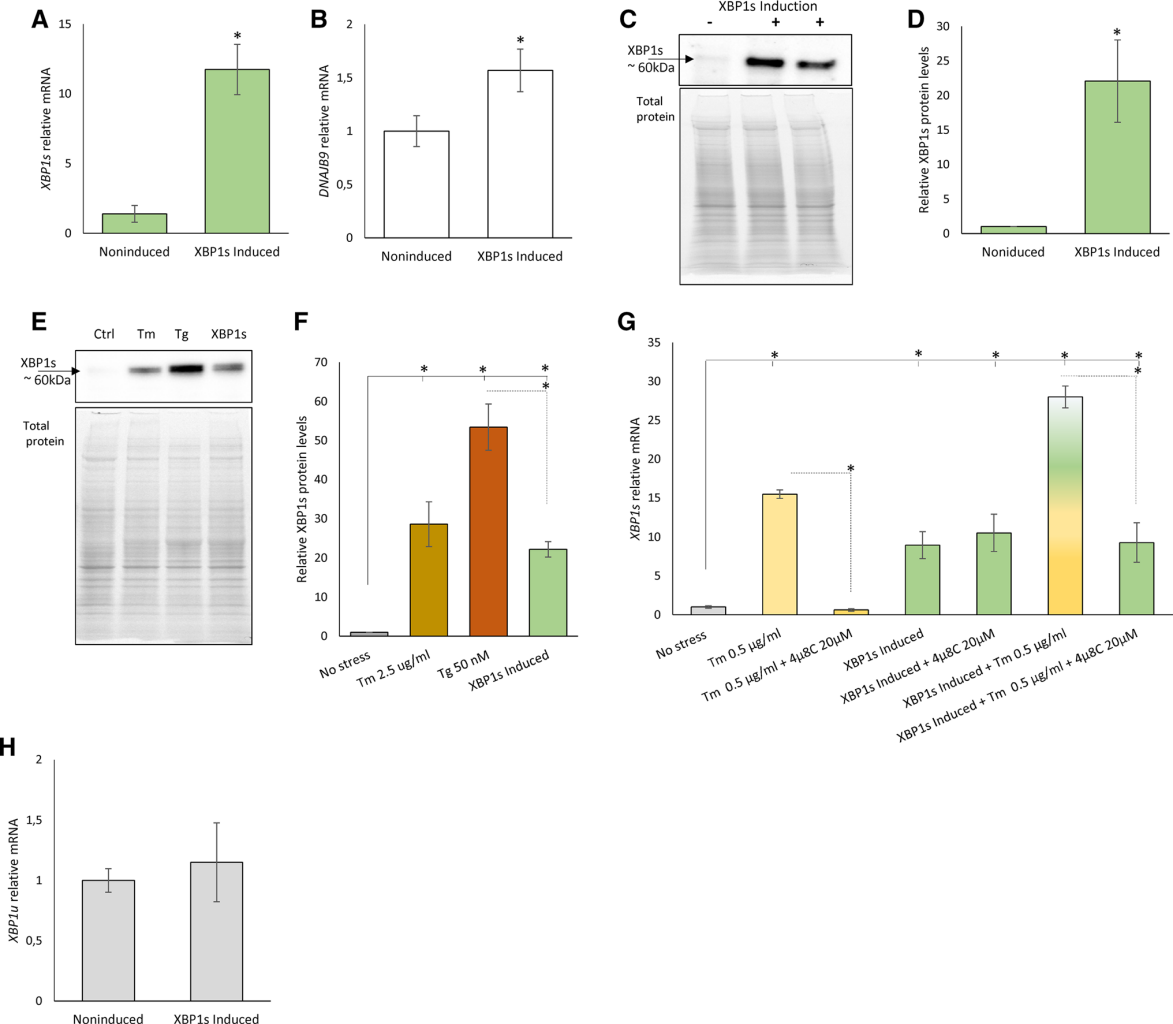
The HeLa-*XBP1s* cell line induced for 24 h accumulates **A** *XBP1* (green) and **B** *DNAJB9* (white) mRNA. The results from three independent experiments (*n* = 9) are plotted normalized to *GAPDH* and *RPLP0* mRNA levels and expressed as a fold change over the noninduced cells. Error bars represent standard deviations. Significant changes (*P *value *P* < 0.05) are marked with an asterisk. The corresponding changes in XBP1 protein levels were evaluated by Western blot (**C**) normalized to total protein levels (**D**) and related noninduced control or evaluated by Western blot and to compared to ER stress induced by Tm (2.5 µg/ml, brown) Tg (50 nM, red) treatment for 6 h (**E**) and related to the no-stress control (**F**). *P < 0.05 was considered significant. **G** The exogenous *XBP1s* mRNA levels were independent IRE1 activity as shown with 4µ8C (20 µM), a specific IRE1 activity inhibitor. The yellow–green bars depict the conditions where XBP1s expression was induced in the presence of Tm and 4µ8C. Tm was used at 0.5 µg/ml concentration for 6 h (yellow). **H** The exogenous *XBP1s* expression does not lead to the accumulation of *XBP1u* mRNA (grey). The results from three independent experiments (*n* = 9) are plotted normalized to *GAPDH* and *RPLP0* mRNA levels and expressed as a fold change over the noninduced cells. Error bars represent standard deviations. Significant changes (*P *value *P* < 0.05) are marked with an asterisk

To follow XBP1’s role in the UPR, we created an inducible HeLa cell line model in which we could express low mRNA levels of this transcription factor that might mimic the levels observed during mild ER stress. Following the expansion and validation of individual clones expressing *XBP1s* under an inducible promoter, we selected a cell line that upon induction, stably expressed about an 11-fold increase *XBP1s* mRNA (HeLa-*XBP1s*) when compared to noninduced cells (Fig. 2A). The obtained XBP1 levels were sufficient to induce the expression of *DNAJB9* mRNA, an XBP1 transcriptional target gene (Fig. 2B). In this case, the *DNAJB9* mRNA was induced only about 1.5-fold, whereas in the corresponding ER stress model the *DNAJB9* expression was higher (about 3–4-fold), suggesting that other transcriptional mechanisms may be responsible for this gene induction during UPR. The levels of *XBP1s* mRNA induction resulted in XBP1 protein levels (Fig. 2C) comparable to those observed after 6 h of ER stress-induced with 2.5 µg/ml Tm and about half of the protein levels observed with 50 nM Tg (Fig. 2 EF), despite the fact that both of these stressors induced *XBP1s* mRNA comparably (Fig. 1C). The exogenous XBP1 signals were independent of IRE1 activity since 4µ8C, a specific IRE1 activity inhibitor, had no effect on the induced *XBP1s* mRNA levels [52] (Fig. 2G). Furthermore, the XBP1 induction did not lead to accumulation of *XBP1u* mRNA (Fig. 2H). Notably, in the presence of doxycycline, the XBP1 protein was stably expressed up to a week (Supplemental Figure S2AB), showing a trend to accumulate after a prolonged time of induction. Finally, the doxycycline used for the induction of *XBP1s* did not induce ER stress (Supplemental Figure S2C) and siRNA knockdown of *XBP1s* mRNA induction with the inducible cell line dramatically reduced the *XBP1s* mRNA levels (Supplemental Figure S2D).

In our analysis of XBP1‐affected factors, we obtained RNA samples from the HeLa-*XBP1s* cell line under control conditions (no induction) and after 24 h of induction, and subjected both to RNA‐seq analysis. Notably in these NGS analysis, inductions of all *XBP1* isoform mRNAs were accessed as a one *XBP1* gene change and in the range of fivefold and that is reflected by about 2 log2 fold change. The isoform-dedicated analysis, however, indicated that the *XBP1s* mRNA induction was about 20-fold. In this analysis, we focused only on genes that were specifically affected by induction by at least a log-fold (twofold change) and had a *P *value below 0.05.

**Fig. 3:**
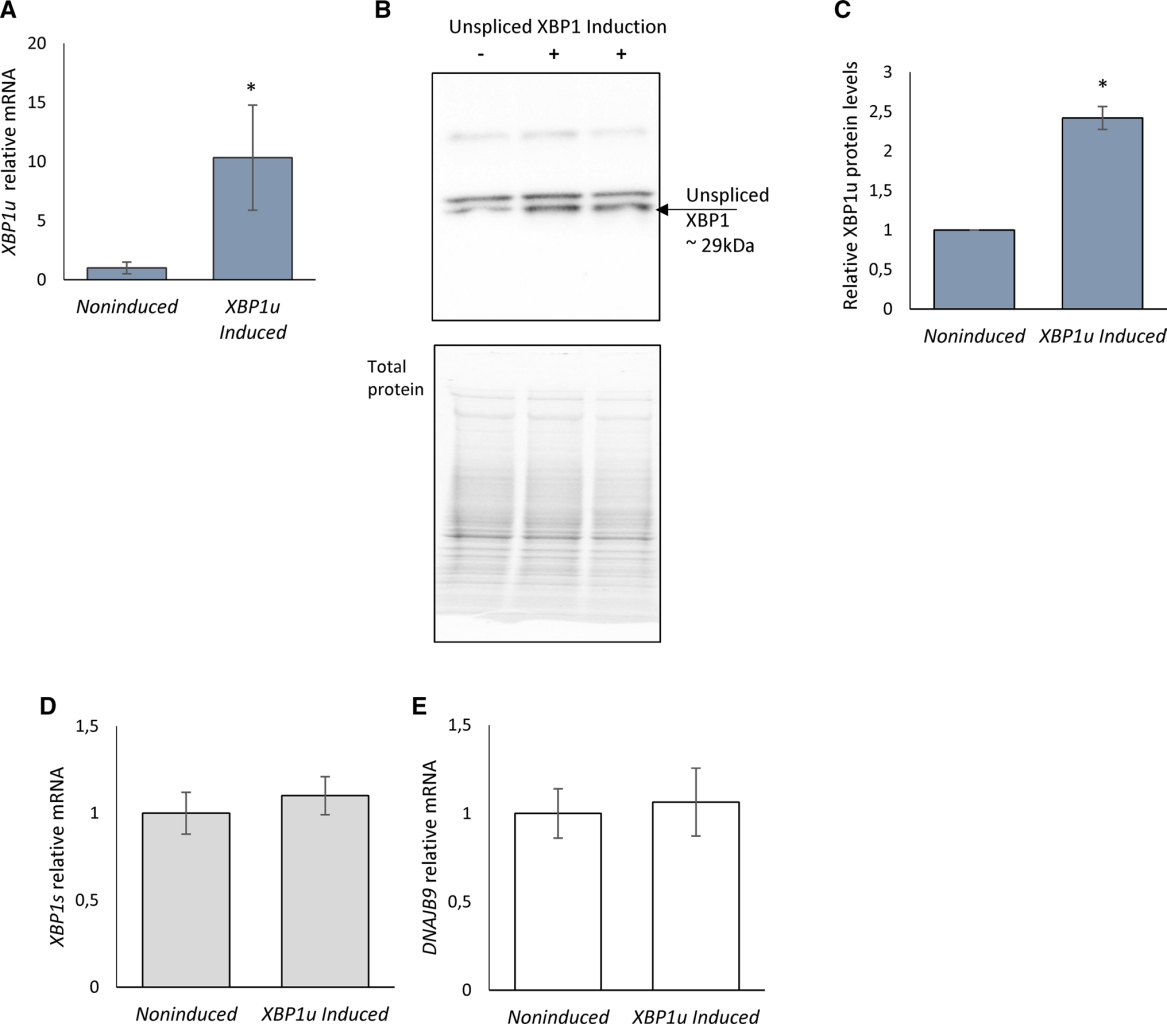
Upon 24-h induction, the HeLa-*XBP1u* cell line accumulates *XBP1u* mRNA. **A** The results from three independent experiments (*n* = 9) are plotted normalized to *GAPDH* and *RPLP0* mRNA levels and expressed as a fold change over the noninduced cells. Error bars represent standard deviations. Significant changes (*P *value *P* < 0.05) are marked with an asterisk. The corresponding changes in unspliced XBP1 protein levels were evaluated by Western blot (**B**) normalized to total protein levels (**C**) and related noninduced control. *P < 0.05 was considered significant. The exogenous *XBP1u* expression does not lead to the accumulation of *XBP1s* (**D**) and *DNAJB9* (**E**) mRNA. The results from three independent experiments (*n* = 9) are plotted normalized to *GAPDH* and *RPLP0* mRNA levels and expressed as a fold change over the noninduced cells. Error bars represent standard deviations. Significant changes (*P *value *P* < 0.05) are marked with an asterisk

We were aware that by applying such “loose” selection parameters, however, would result in a large group of genes. Given this concern, we performed independent validations of the XBP1 predicted targets. Furthermore, given the large number of identified genes fulfilling this criterion could also result from the doxycycline treatment. As a control, therefore, the RNA-seq data obtained from a comparable inducible HeLa cell line expressing low levels of *XBP1u* (HeLa-*XBP1u*) were examined. These induced genes were omitted from further analysis. As previously mentioned, *XBP1s* mRNA results from ER stress-activated IRE1 splicing of a 26 nt unconventional intron in the coding region of unspliced *XBP1* (*XBP1u)* that causes a frameshift. The XBP1 protein (~ 48 kDa) has the same N-terminus, but a longer and distinct C-terminus compared to the unspliced XBP1 protein (~ 29 kDa) [5, 53]. More importantly, the new C-terminus in XBP1 contains the transactivation domain [5, 53]. The induction levels of *XBP1u* mRNA in these cells were in the tenfold range that did result in unspliced XBP1 protein expression (Fig. 3A–C) but did not result in *XBP1s* mRNA accumulation (Fig. 3D) or any increase in XBP1 transcriptional activity (Fig. 3E) or protein (Supplemental Figure S1B). The unspliced XBP1 protein levels are in agreement with previous reports that this protein is rapidly degraded [54]. Furthermore, *XBP1u* induction was performed under no ER stress conditions to avoid the possibility of a negative impact of unspliced XBP1 protein on spliced XBP1 [55]. Given this, the RNA-seq data obtained from the induced HeLa-*XBP1u* cell line were an appropriate control for our experiments.

**Fig. 4:**
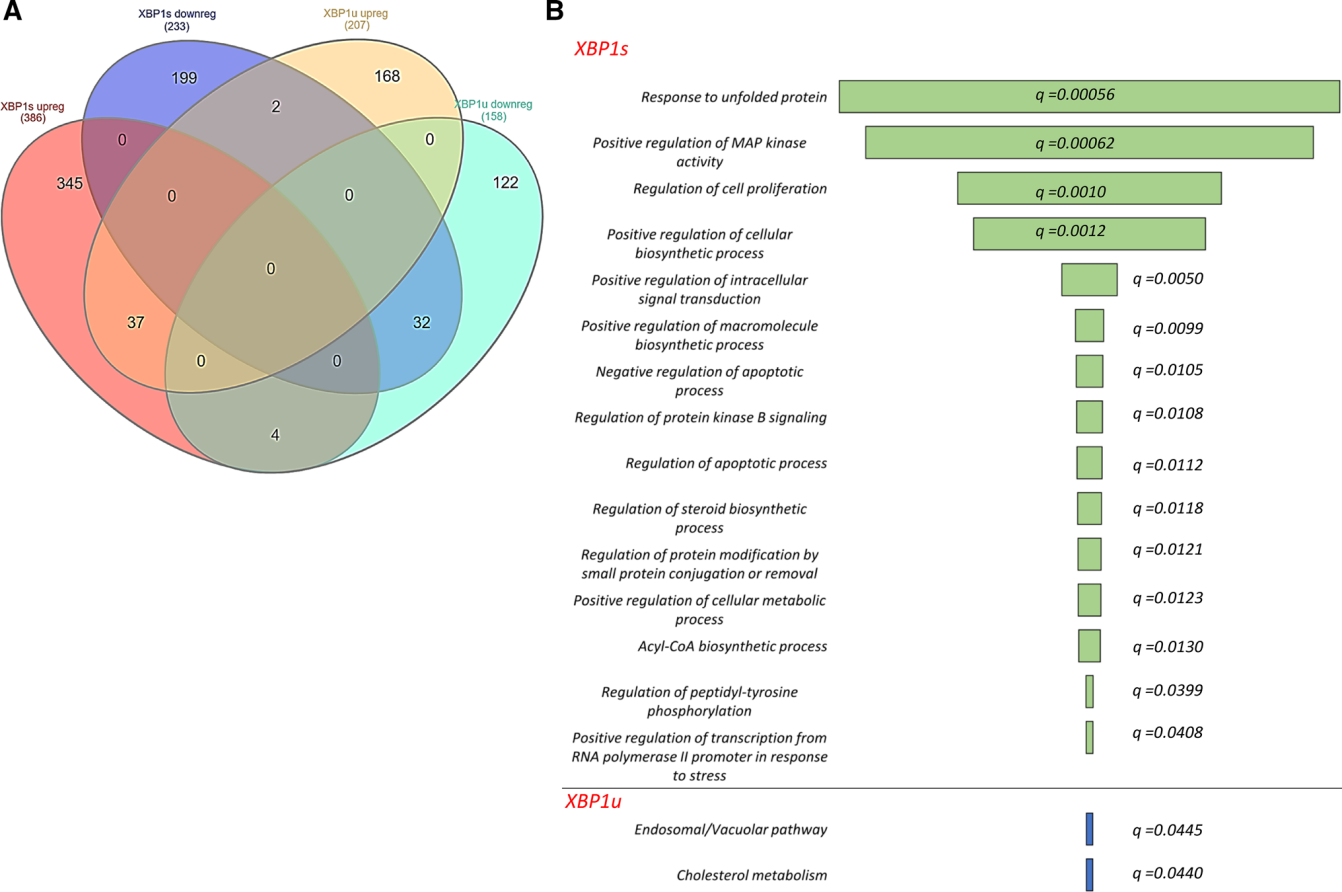
The impact of the exogenic *XBP1u* and *XBP1s* induction on HeLa genome-wide cellular mRNA profiles and their potential functional consequences. **A** The Venn diagram [56] represents the general distribution of mRNAs that were significantly (P < 0.05) affected by *XBP1s* and *XBP1u* transgenes—Supplemental Table 2C. **B** Transcripts reduced and induced upon XBP1 induction are marked with dark blue and red, respectively, whereas mRNAs reduced and induced upon unspliced XBP1 induction are marked with light blue and yellow, respectively. The Gene Ontology assignment of the cellular functions of mRNAs potentially regulated by the spliced XBP1 (XBP1s) or unspliced XBP1 (XBP1u) as assigned by the Enrichr Web server—Supplemental Table 2D [40]. The green bar color depicts the *q* value less than 0.05. The longer bars have the lower *q* values

The overlap of the RNA-seq data obtained from induced spliced XBP1 and unspliced XBP1 expression systems is illustrated in a Venn diagram in Fig. 4A. The functional assignment of XBP1 modulated genes was performed with Enrichr Web server using a strict criterion (*P *value *P* < 0.05 and *q *value *q* < 0.05). As shown in Fig. 4B, this analysis of expression profiles specific for cells expressing *XBP1u* did not revealed any specific activation of ER stress, UPR signaling or apoptotic pathways. Nevertheless, some of the unspliced XBP1-related expression changes could be assigned to cholesterol metabolism and the endosomal pathway. Furthermore, analysis of genes that were common between spliced and unspliced *XBP1* mRNA expression profiles did not result in any significant functional assignment. Notably, because of *XBP1s* induction, the mRNA of 345 and 199 genes were induced or reduced, respectively. Furthermore, this expression profile correlated well with changes related to the UPR that included protein folding, cellular proliferation, and negative regulation of apoptotic pathways (Fig. 4B).

**Fig. 5:**
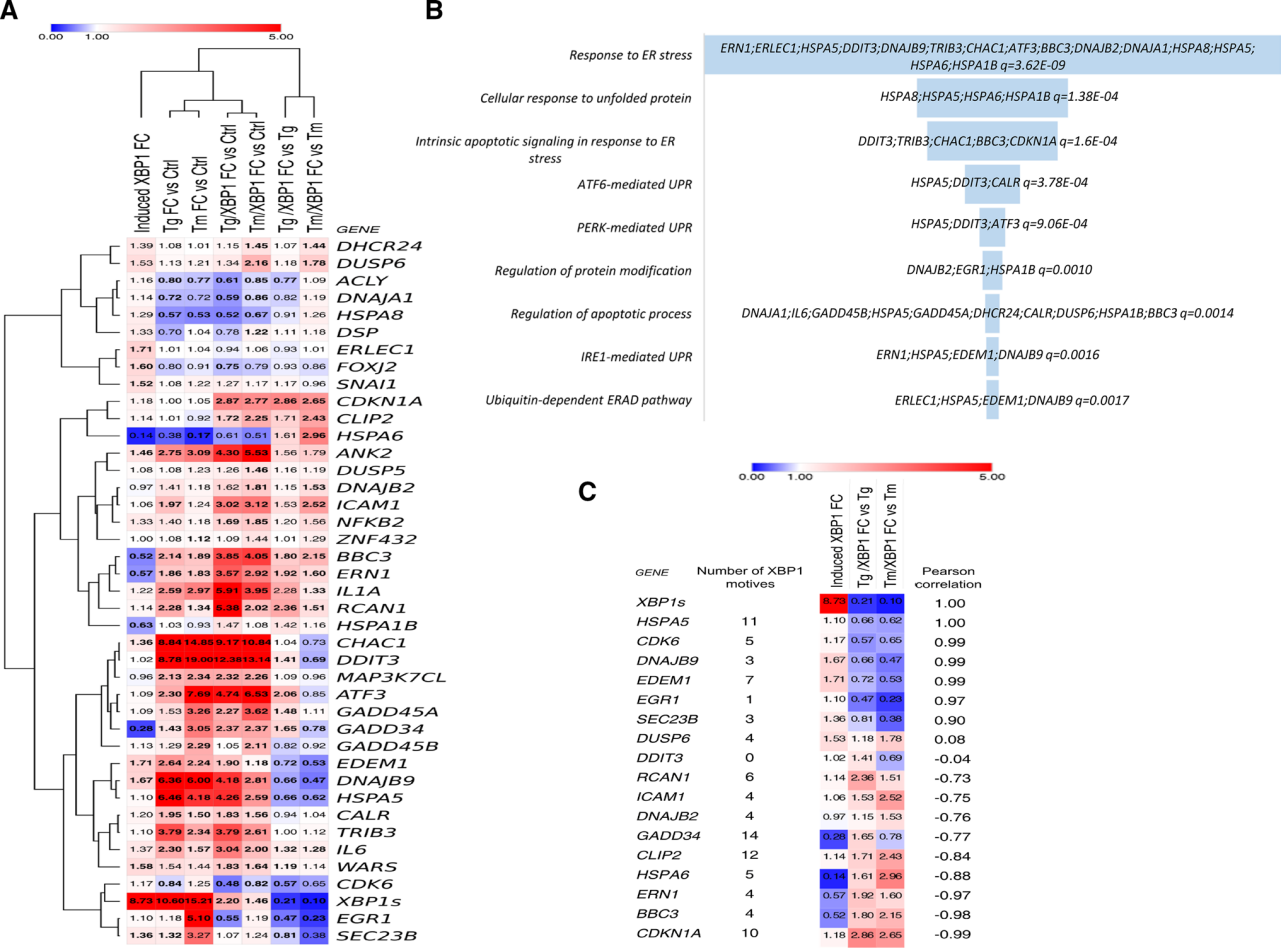
The heat map representing expression changes of all verified potentially XBP1-dependent genes identified in induced *XBP1s* expression experiments (Induced XBP1) and separately in silencing *XBP1* during mild stress experiments in HeLa cells (Tg/XBP1s and Tm/XBP1s). **A **The logo of XBP1-binding motif M00402 (consensus: GACGTGkCmtww, where *k* = *G* or *T*; *m* = *A* or *C*; *w* = *A* or *T*) from the Hocomoco v. 9 motif library. **B** Heat maps were generated and organized according to hierarchical clustering of expression changes with the Morpheus software (Morpheus, https://software.broadinstitute.org/morpheus). **C** FC depicts “fold change”, Tm—tunicamycin 0.5 µg/ml induced ER stress for 6 h, Tg—thapsigargin 2.5 nM induced ER stress for 6 h, whereas Tm/XBP1 S and Tg/XBP1 S depict *XBP1* silencing in each of these stress models. **D** The Enrichr Web server (https://amp.pharm.mssm.edu/Enrichr/) was applied to assign the qPCR results into the “Gene Ontology Biological Process” categories with a selection based on a *q *value *q* < 0.05. Heat map representing the mRNAs that display the most correlated changes with *XBP1s* mRNA levels as calculated by Pearson correlations

Given that XBP1’s impact on gene expression can result from both direct and indirect effects with transcription factors originating from the other UPR branches (PERK and ATF6), we decided to narrow our verification set to the genes that contained potential promoter regions for *XBP1*. To verify the potential direct role of XBP1 during the UPR, the identified gene locations were analyzed for the presence of XBP1-binding motifs (Fig. 5A). Our analysis was in HeLa cells, and we focused only on transcriptionally active chromatin regions.

This resulted in the selection of 58 genes potentially directly regulated by spliced XBP1 that were then validated in 3 independent biological replicates using 96-well qPCR arrays (Supplemental Table 2AB). The expression changes were accessed following XBP1 overexpression as well as silencing with specific siRNA against *XBP1* during 6 h of mild ER stress (induced with Tm or Tg). Furthermore, in our validation set, we included *HSPA5* (*BiP*) and *DDIT3* (*CHOP*) mRNAs that were previously also reported to be regulated by XBP1 [57]. Interestingly, the *CHOP* region did not contain any potential XBP1-binding motifs, whereas in *BiP* there were 11 sites. This approach identified significant changes in 40 of the transcripts (Table 1).

**Table 1:** Summary of mRNA changes observed during *XBP1s* induction, ER stress-induced with Tg or Tm, and Tg or Tm ER stress together with *XBP1* silencing (*Tg* and Tm/*XBP1* S)

Gene	Number of XBP1 motifs	Induced *XBP1s* FC	Tg FC vs Ctrl	Tg/XBP1 S FC vs Ctrl	Tg /XBP1 S FC vs Tg	Tm FC vs Ctrl	Tm/XBP1 S FC vs Ctrl	Tm/XBP1 S FC vs Tm
*ACLY*	1	1.15	**0.80**	**0.61**	**0.77**	**0.77**	**0.85**	1.09
*ANK2*	1	**1.45**	**2.75**	**4.30**	1.56	**3.09**	**5.53**	1.79
*ATF3*	6	1.09	**2.30**	**4.74**	**2.06**	**7.69**	**6.53**	0.85
*BBC3*	4	**0.51**	**2.14**	**3.85**	**1.80**	**1.89**	**4.05**	**2.15**
*CALR*	14	1.19	**1.95**	**1.83**	0.94	**1.50**	**1.56**	1.04
*CDK6*	5	1.17	**0.84**	**0.48**	**0.57**	1.25	**0.82**	0.65
*CDKN1A*	10	1.18	1.00	**2.87**	**2.86**	1.05	**2.77**	**2.65**
*CHAC1*	1	**1.36**	**8.84**	**9.17**	1.04	**14.85**	**10.84**	0.73
*DDIT3*	0	1.02	**8.78**	**12.38**	**1.41**	**19.00**	**13.14**	**0.69**
*CLIP2*	12	1.13	1.01	**1.72**	1.71	0.92	**2.25**	**2.43**
*DHCR24*	2	1.39	1.08	1.15	1.07	1.01	**1.45**	**1.44**
*DNAJA1*	4	1.13	**0.72**	**0.59**	0.82	0.72	**0.86**	1.19
*DNAJB2*	4	0.96	1.41	1.62	1.15	1.18	**1.81**	**1.53**
*DNAJB9*	3	**1.67**	**6.36**	**4.18**	**0.66**	**6.00**	**2.81**	**0.47**
*DSP*	2	1.33	0.70	0.78	1.11	1.04	**1.22**	1.18
*DUSP5*	12	1.08	1.08	1.26	1.16	1.23	**1.46**	1.19
*DUSP6*	4	1.52	1.13	1.34	1.18	1.21	**2.16**	**1.78**
*EDEM1*	7	**1.71**	**2.64**	**1.90**	**0.72**	**2.24**	**1.18**	**0.53**
*EGR1*	1	1.09	1.18	**0.55**	**0.47**	**5.10**	1.19	**0.23**
*ERLEC1*	6	**1.70**	1.01	0.94	0.93	1.04	1.06	1.01
*ERN1*	4	**0.56**	**1.86**	**3.57**	**1.92**	**1.83**	**2.92**	**1.60**
*FOXJ2*	4	**1.59**	0.80	**0.75**	0.93	0.91	0.79	0.86
*GADD45A*	2	1.08	1.53	**2.27**	**1.48**	**3.26**	**3.62**	1.11
*GADD45B*	2	1.12	1.29	1.05	0.82	**2.29**	**2.11**	0.92
*HSPA1B*	13	**0.63**	1.03	1.47	1.42	0.93	1.08	1.16
*HSPA5*	11	1.10	**6.46**	**4.26**	**0.66**	**4.18**	**2.59**	**0.62**
*HSPA6*	5	0.13	0.38	0.61	1.61	**0.17**	0.51	2.96
*HSPA8*	1	1.29	**0.57**	**0.52**	0.91	**0.53**	**0.67**	1.26
*ICAM1*	4	1.06	**1.97**	**3.02**	1.53	1.24	**3.12**	**2.52**
*IL1A*	3	1.21	**2.59**	**5.91**	2.28	**2.97**	**3.95**	**1.33**
*IL6*	4	1.37	**2.30**	**3.04**	**1.32**	**1.57**	**2.00**	**1.28**
*MAP3K7CL*	4	0.95	**2.13**	**2.32**	1.09	**2.34**	**2.26**	0.96
*NFKB2*	11	1.32	1.40	**1.69**	1.20	1.18	**1.85**	**1.56**
*RCAN1*	6	1.13	**2.28**	**5.38**	**2.36**	**1.34**	**2.02**	**1.51**
*SEC23B*	3	**1.36**	**1.32**	1.07	**0.81**	3.27	1.24	0.38
*SNAI1*	5	**1.51**	1.08	1.27	1.17	1.22	1.17	0.96
*TRIB3*	5	1.10	**3.79**	**3.79**	1.00	**2.34**	**2.61**	1.12
*WARS*	3	**1.58**	1.54	**1.83**	**1.19**	1.44	**1.64**	1.14
*GADD34*	14	**0.27**	**1.43**	**2.37**	**1.65**	**3.05**	**2.37**	**0.78**
*ZNF432*	3	1.01	1.08	1.09	1.01	1.12	1.44	1.29
*XBP1s*		8.73	10.60	2.20	0.21	15.21	1.46	0.10

As shown in Table 1 and Supplemental Table 2A, we observed that following *XBP1s* induction, 8 mRNAs were significantly induced that included a*nkyrin 2* (*ANK2*), *glutathione-specific gamma-glutamylcyclotransferase 1* (*CHAC1*), *ER-Resident Protein ERdj4*,* DNAJB9*, *ER degradation-enhancing alpha-mannosidase-like protein 1* (*EDEM1*), *ER lectin 1* (*ERLEC1*), *forkhead box J2* (*FOXJ2*), *SEC23B*, and *interferon-induced protein 53* (*WARS*). Notably, *ERLEC1*,* WARS* and *SEC23B* have been previously identified as XBP1-dependent genes in MCF-7 and HEK-293 cells [58, 59], whereas *CHAC1* has been identified recently as an important regulator of UPR-associated ferroptosis [60]. Furthermore, 5 mRNAs were significantly reduced upon *XBP1s* induction and included (1) *IRE1 (ERN1)*, (2) *growth arrest and DNA damage-inducible protein* (*GADD34*) [61], a crucial PERK pathway regulator, (3) *PUMA (BBC3)*, an important ER stress-related proapoptotic factor [62]*)*, (4) *heat shock protein family A (Hsp70) member 1B* (*HSPA1B*), and (5) *h*e*at shock protein family A (Hsp70) member 6* (*HSPA6*)*.*

In the parallel *XBP1*-silencing experiments (Table 1 and Supplemental Table 2B), we observed that mRNA levels of well-known XBP1 targets such as *DNAJB9* and *EDEM1* [44, 57] as well as interleukin 6—*IL6* [63] were significantly induced by both stressors and reduced upon *XBP1* silencing. In addition, as previously reported, *XBP1* silencing had a limited effect on *HSPA5* (BiP) expression during ER stress [57]. Importantly, we also observed that *ERN1*, *BBC3*, and regulator of calcineurin 1 (*RCAN1*) were induced by both stressors and their levels were even higher after *XBP1* silencing. Notably, we recently identified *RCAN1* as an important prosurvival regulator of ER stress-induced cell fate decisions [21].

We also noted the induction of ER stress proapoptotic c*yclin-dependent kinase inhibitor 1A* (*CDKN1A (p21*)) and cytoplasmic linker 2 (*CLIP2*) expression upon *XBP1* silencing. Interestingly, *CLIP2* has been associated as a key gene for diabetes mellitus development [64]. The PERK-dependent cyclin-dependent kinase 6 (*CDK6* [65]) expression was significantly reduced only by Tg and its levels became even lower upon *XBP1* silencing during both Tm and Tg treatments. *ANK2*,* ATF3* and *GADD45A* mRNAs were induced by both stressors and their levels were further elevated upon *XBP1* silencing during Tg treatment. Notably, the UPR-induced proapoptotic *GADD45A* [21] has been proposed as an XBP1-dependent gene [57]. Furthermore, *HSPA6* mRNA levels were significantly induced upon *XBP1* silencing in Tm- and Tg-treated cells, but the expression of the *HSPA6* gene was exceptionally low in HeLa cells, however, and therefore, this observation will require further verification.

We also observed that *XBP1* silencing resulted in lower expression of the transcription factor involved in ER stress-related regulation of cell cycle progression *early growth response 1*—*EGR1* [66] in both stress models. In addition, *XBP1* silencing induced the anti-apoptotic *dual-specificity phosphatase 6—DUSP6* gene [67] expression in Tm-treated cells. Notably, *DDIT3* (*CHOP*) and *GADD34* levels were significantly induced in both ER stress models, and further increased upon *XBP1* silencing in Tg-treated cells whereas they were reduced in Tm-treated cells (Fig. 5A). Furthermore, we did not find any significant correlation between the potential number of XBP1-binding motifs and their connection to actual functional effects. Importantly, the functional analysis of all these verified XBP1-related genes indicated that they were almost exclusively connected to ER stress outcomes including all three UPR branches and ERAD, and the regulation of apoptotic processes (Fig. 5B).

Despite some discrepancies between the data obtained from the *XBP1* induction and silencing in different ER stress models, we were able to define a group of genes whose expression was XBP1 dependent, and their expression was significantly affected in at least two out of three independent experimental approaches (XBP1 induction; silencing *XBP1s* during Tm treatment; or silencing *XBP1s* during Tg treatment) (Table 1). As shown in Fig. 5C, this resulted in a final selection of a group of 17 genes. In this gene set, the expression of six genes (*HSPA5*, *CDK6*, *DNAJB9*, *EDEM1*, *EGR1*, and *SEC23B*) was positively correlated with the XBP1 levels.

**Fig. 6:**
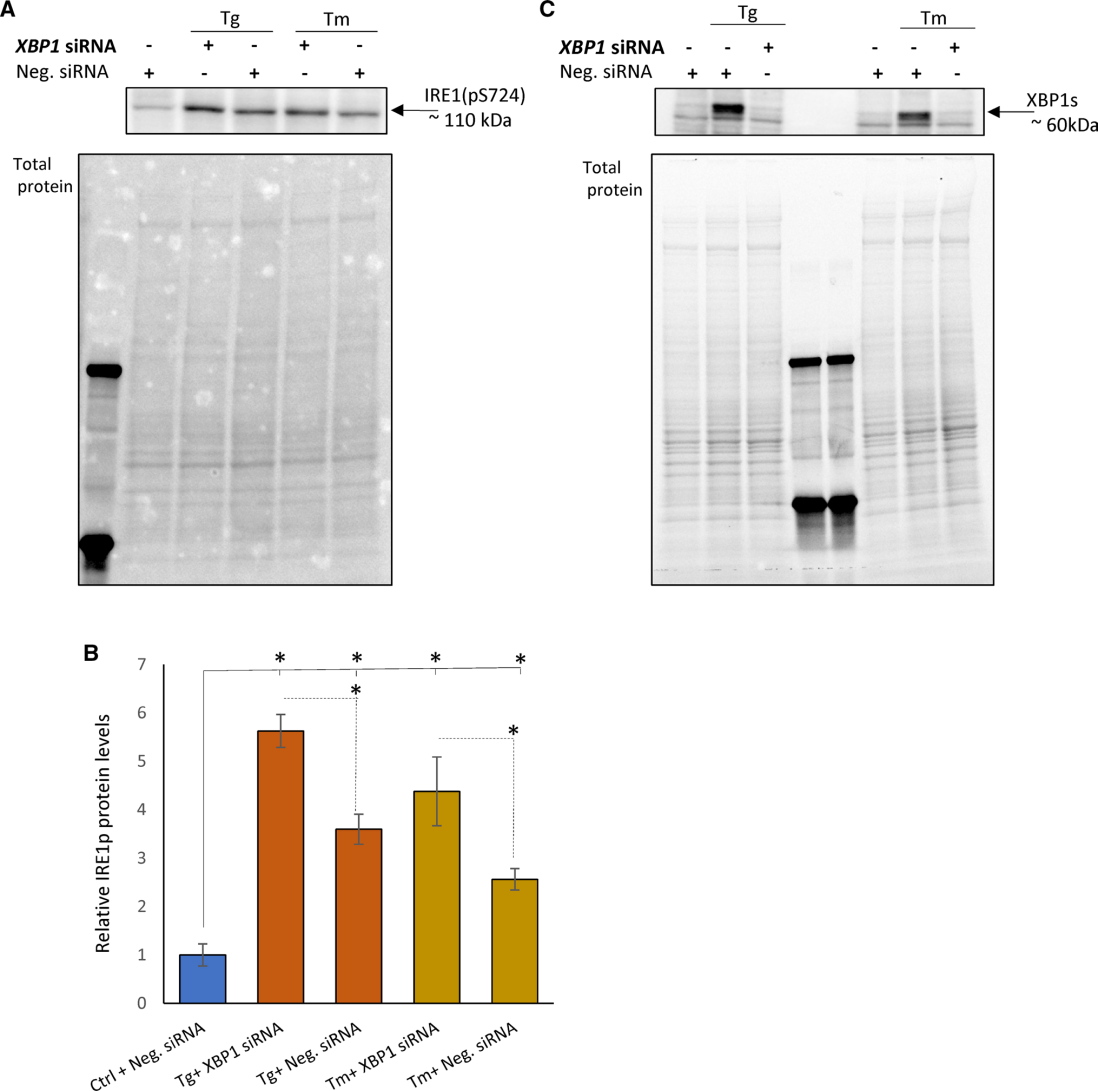
The *XBP1s* silencing is accompanied by reduced IRE1p protein levels in Tg- and Tm-treated HeLa cells. **A** Representative changes in IRE1p protein levels in HeLa cells treated with Tg (50 nM) and Tm (2.5 µg/ml) and Tg for 6 h in the presence or absence of a specific siRNA against *XBP1* as evaluated by Western blot and **B** normalized to total protein levels and related to the no-stress control. The experiments were repeated three times. Error bars represent standard deviations and *P < 0.05 was considered significant. **C** The corresponding changes in XBP1 protein levels were evaluated by Western blot

Notably, we also identified three genes, *BBC3*, *ERN1* and *HSPA6*, whose expression was negatively correlated with the *XBP1* expression in both ER stress models. Given that XBP1-mediated attenuation of *ERN1* expression suggests a novel negative-feedback regulatory loop between XBP1 and IRE1, we also tested if XBP1-related reduction of *ERN1* mRNA levels were reflected by IRE1 protein changes in Tm- and Tg-treated cells. In HeLa cells exposed to both Tm and Tg, the phosphorylated IRE1 protein levels were increased upon *XBP1* silencing as shown in Fig. 6.

**Fig. 7:**
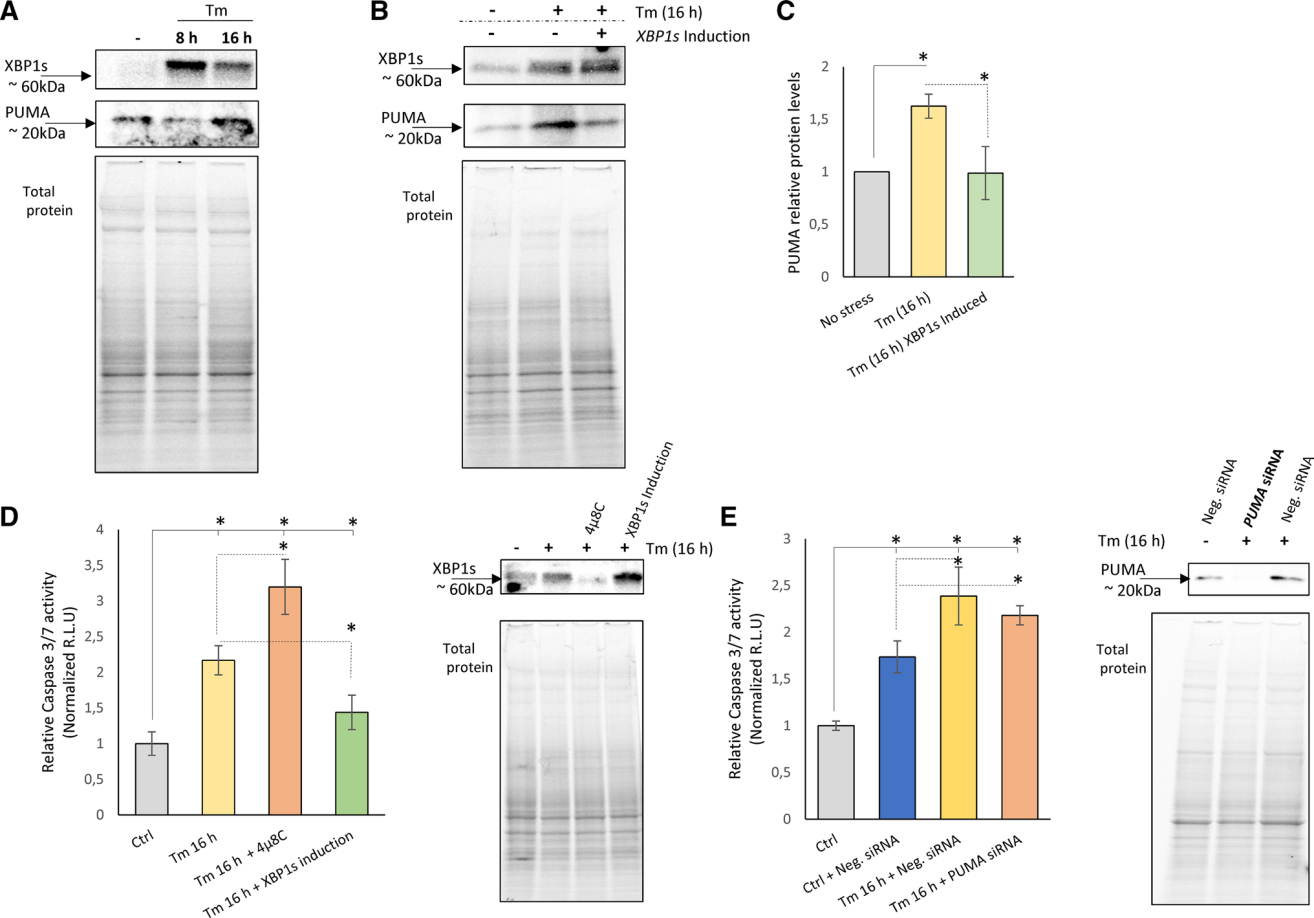
The 24 h induction of *XBP1s* is accompanied by reduced PUMA protein levels and lower Caspase 3/7 activity in Tm-treated HeLa cells. **A** Representative changes in XBP1 and PUMA protein levels in HeLa cells treated with Tm (2.5 µg/ml) for 8 and 16 h as evaluated by Western blot. **B** The changes in XBP1 and PUMA protein levels in *XBP1s*-induced and -noninduced HeLa cells treated with Tm (2.5 µg/ml) 16 h were evaluated by Western blot (**C**) normalized to total protein levels and related to the noninduced, no-stress control, and *P < 0.05 was considered significant. *XBP1s*-induced (24 h) and -noninduced HeLa cells were treated with Tm (2.5 µg/ml) for 16 h in the presence or absence of 4µ8C (20 µM), a specific IRE1 activity inhibitor. **D** The caspase 3/7 activity was monitored by luminescence and expressed in relative light units (RLU). Cells for each time point were seeded in triplicate, and the experiments were repeated three times. Error bars represent standard deviations and *P < 0.05 was considered significant. The corresponding changes in XBP1 protein levels were evaluated by Western blot. **E** HeLa cells were treated with Tm (2.5 µg/ml) for 16 h in the presence or absence of a specific siRNA against *BBC3 (PUMA)*. The caspase 3/7 activity was monitored by luminescence and expressed in RLU. Cells for each time point were seeded in triplicate, and the experiments were repeated three times. Error bars represent standard deviations, *P < 0.05 was considered significant. The corresponding changes in PUMA protein levels were evaluated by Western blot

Given that *BBC3*-encoded PUMA protein was reported as an important apoptotic factor during UPR, we also tested if XBP1-related reduction of *BBC3* mRNA levels were reflected by PUMA protein changes. As shown in Fig. 7A, in HeLa cells exposed to Tm (2.5 µg/ml), PUMA protein levels were negatively correlated with *XBP1s* expression and reduced after 8 h when the XBP1 expression is maximal. Furthermore, when XBP1 is reduced after 16 h of treatment, the *PUMA* levels rise. To support the idea of the negative correlation, we performed an experiment to test the idea of maintaining XBP1s levels by keeping transgenic XBP1s levels high at 16 h during Tm treatment to see the effect on PUMA expression (Fig. 7B, C). The results clearly show that higher XBP1s levels prevented PUMA accumulation during Tm ER stress at 16 h, confirming XPB1s repression on PUMA expression.

To test XBP1’s impact on prosurvival UPR activity, we subjected HeLa cells to Tm-induced ER stress for 16 h and measured caspase 3/7 activity. We directly compared Tm-induced ER stress to Tm-induced stress in the presence of IRE1 inhibitor (4µ8C) which would prevent IRE1 RIDD activity during *XBP1s* mRNA formation. We found that impairing IRE1 activity accelerates cell death in ER-stressed cells, whereas XBP1s expression correlated with a clear prosurvival effect (Fig. 7D). It has been previously reported that upon XBP1 deficiency, IRE1 switches to perform RIDD [68]. Nevertheless, given that the *PUMA* mRNA sequence does not contain the *IRE1-*required consensus sequence (CUGCAG) [69], it seems unlikely that this transcript is degraded by RIDD. Furthermore, inhibiting this IRE1 activity can also prevent death receptor 5 (*DR5*) mRNA degradation and thus apoptosis engagement via caspase-8 [16]. Finally, since PUMA was reported to stimulate the intrinsic pathway of apoptosis [70, 71], we performed an analogous experiment and siRNA silenced *PUMA* (Fig. 7E). Interestingly, we did not observe any significant changes in caspase 3/7 activity related to *PUMA* depletion (Fig. 7E). PUMA or BH3 protein **(**Bid) can separately activate Bax [72, 73], and this could potentially lead to mitochondrial outer membrane permeability and apoptosis [74]. In the absence of PUMA, other BH3-only proteins (Bid and Bim) that are PERK dependent [75, 76] could still be efficiently activating caspase 3/7.

To generalize the findings to other cell types, we followed consequences of *XBP1* silencing on both *ERN1* and *BBC3* mRNA levels in several other human cell lines exposed to ER stress that included Schwann Cells (SNF96.2), epidermal keratinocytes (HaCaT), umbilical vein endothelial cells (HUVEC) and bronchial epithelial cells (16HBE14o-). As shown in Supplemental Figure S3, BBC3 expression was significantly induced upon *XBP1* silencing in all of these experimental models. Furthermore, *ERN1* levels were modestly but significantly higher in the absence of XBP1s in all Tg-treated cell lines as well in HaCaTs and HUVECs treated with Tm (Supplemental Figure S3). These data support the general repressive role of XBP1 on *ERN1* levels in a diverse group of human cell lines*.*

In summary, we identified eight genes whose expression was ER stress model-dependent and/or not affected by XBP1 induction alone: *DUSP6*, *DDIT3*, *GADD34*,* RCAN1*, *ICAM1*, *DNAJB2*, *CLIP2* and *CDKN1A*. This set contains mainly genes that can be attributed to the PERK pathway activity (*DDIT3*, *GADD34*) and cell survival (*DDIT3*, *GADD34*, *RCAN1* and *CDKN1A*), suggesting that their levels result from both XBP1 activity and other UPR pathways, including the PERK pathway.

## Discussion

The IRE1-dependent pathway is the most ancient and conserved branch of UPR [25] and serves as the molecular timer and executor for ER stress-related cell death [77–79]. It is, therefore, not surprising that the role of its downstream transcription factor, XBP1, has been extensively studied [44, 49, 57–59, 80, 81]. The transcriptional targets of XBP1 are well identified and consist of ERAD components (*EDEM1*), chaperones (*HSPA5*, *DNAJB9*, and *DNAJC3*), and vesicle-trafficking components (*SEC23B*) [57–59, 80]. Furthermore, genes involved in the inflammatory responses (including *IL6*) [59], as well as genes not related to UPR pathways including adipocyte and myogenic differentiation (*C/EBP* and *MIST1*) have been proposed as tissue-dependent XBP1 transcriptional targets [82]. Consequently, the XBP1 has been widely accepted as an adaptive component of UPR that is responsible for facilitating protein folding and ER expansion.

Despite these advances, determining the global network of XBP1 transcriptional activity and its consequences on cell fate decisions remains less clear. Part of the problem in defining XBP1’s role involves analyzing its function in models that are often based on one type of ER stressor or are utilizing high levels of overexpression of XBP1. Furthermore, the studies often focus on a small subset of induced genes. In our approach, we exploited inducible cell lines capable of *XBP1(s)* and *XBP1(u)* lower expression levels and two models of pharmacological ER stress induction, glycosylation inhibition and disruption of ER calcium homeostasis. Using this system, we were able to demonstrate the induction of the main UPR mediators including *HSPA5* (*BIP*), *IRE1*, *XBP1s* and *CHOP*. Notably, the levels of *XBP1s* mRNA obtained during cell line induction were on the low end of those observed in our ER stress models.

To follow the XBP1-related changes in transcriptome, we performed next-generation sequencing profiling in HeLa cells with the induced expression of *XBP1s* or *XBP1u* and focused on changes in gene expression related mainly to prosurvival and apoptotic UPR signaling pathways. Notably *XBP1u* expression did not result in any changes in which we could clearly assign to these activities. The unspliced XBP1 protein has been shown to be rapidly degraded and maintained at low levels and the *XBP1u* transgene transcript could not be processed to functional XBP1, and our results are in good agreement with previous reports [54].

Nevertheless, the *XBP1s* transgene induction resulted in wide changes of expression profiles of the genes involved in the UPR including *DNAJB9* and *EDEM1*, stress responses, and regulation of cellular biosynthetic and apoptotic responses (Fig. 4). The results confirmed XBP1’s role as a crucial UPR mediator and potentially defined a large set of genes which resulted from XBP1 transcriptional activity. Importantly, following *XBP1s* induction, we did not observe some of the classical UPR activation genes since both *BIP* and *CHOP* mRNA levels remained relatively constant, and therefore, it is quite plausible that the observed transcriptomic changes often seen did not result specifically from XBP1 activity.

To further test this hypothesis, we selected a set of 58 genes **(**Supplemental Table 2) whose genomic locations were in the proximity of XBP1-binding motifs and *DDIT3* and *HSPA5* and validated them independently. The results revealed XBP1-related changes in 40 transcripts, most of which were related to the UPR stress responses and regulation of apoptosis (Fig. 5). The number of potential XBP1-binding motifs in the promoter regions of genes did not correlate well with their transcript expression levels, suggesting that other requirements such as the relative position from the transcriptional start site or the presence of other potential binding motifs (such as ATF6 for example [57, 59]) may be necessary to achieve efficient transcription. However, the effects of the number of transcription factor-binding motifs on expression are only observed for some transcription factors [83]. Nevertheless, homotypic clusters of motifs for some transcription factors are known to potentiate the effects of these factors on expression for some genes [84]. Therefore, we also tested our gene set for a correlation between XBP1 motif clusters and fold changes, but no significant effects were observed. Finally, taking into account extreme complexity and dynamic course of UPR signaling, it is important to note that XBP1 cooperates with the other arms of UPR to modulate transcriptomic profiles, rather than being a master regulator of gene expression. Nevertheless, most of the preselected genes displayed expression patterns that positively correlated with the elevated expression levels of XBP1.

Despite our best efforts to find the optimal mild ER stress conditions, Tm and Tg still have different effects on the course of the UPR signaling pathways. For example, the Tm-treated cells were more prone to apoptosis (Fig. 1). We suggest that these differences between Tm and Tg could result in differences in the expression profiles of the cell fate decisions and the PERK-induced genes that include *DDIT3*, *GADD34*, *ATF3* and *RCAN1*. In general, silencing *XBP1s* during Tg treatment resulted in higher expression of *DDIT3*, *GADD34*, *ATF3* and *RCAN1*, whereas a complete lack of XBP1 during Tm treatment resulted in reduced expression of these genes. Since all these transcripts are closely related to the PERK branch of UPR, there exists the intriguing possibility of XBP1-mediated crosstalk between this pathway and the IRE1 branch that determines cell fate decisions [85, 86]. This hypothesis is also supported by the observation that inhibiting the PERK arm of the UPR has a different impact on *XBP1s* mRNA levels in cells treated with Tm when compared to cells treated with Tg (Supplemental Figure S4). All these genes are also potentially regulated by the other UPR-related transcription factors including CHOP and ATF6 (Supplemental Table S3). Hence, our results suggest that the gene expression modulations by XBP1 can also be influenced by other UPR pathways. This hypothesis, however, and the related mechanisms controlling this will obviously require further study.

Despite the differences between the data obtained from the XBP1 induction and silencing in different ER stress models, we were able to define a group of genes whose expression was clearly XBP1 dependent (Fig. 5C). The expression of *HSPA5*, *CDK6*, *DNAJB9*, *EDEM1*, *EGR1*, and *SEC23B* was clearly positively correlated with the XBP1 levels. In all these genes, their expression was not only induced along with *XBP1s* induction, but it was also reduced when *XBP1* was silenced in both stress models. However, *HSPA5*, *EGR1* and *CDK6* only correlate positively in our ER stress-induced conditions and not in an *XBP1* overexpression model, suggesting that ATF6 may also be required for their expression induction [57, 59, 87].

Here, we have identified the 3 genes, *BBC3 (PUMA)*, *ERN1 (IRE1)* and *HSPA6 (BiP)*, whose expression was clearly negatively correlated with the XBP1, and their levels were reduced upon *XBP1* induction and induced upon *XBP1* silencing in both stress models. In the case of *HSPA6*, the expression levels in HeLa cells were extremely low, and therefore, may represent a cell-type-specific effect. The expression levels for the *BBC3* and *ERN1*, however, were more robust and clearly indicated this reversal effect during siRNA silencing of *XBP1s*. Obviously, both *BBC3 (PUMA)* and *ERN1 (IRE1)* are crucial UPR regulators, and their XBP1-dependent repression reveals a novel regulatory mechanism in the UPR.

The ability of XBP1 to attenuate *ERN1* expression and thus reduce IRE1 activity identifies novel negative-feedback regulatory loop between XBP1 and IRE1. Although this observation and potential consequences of such a regulation requires further verification, it is clear that other UPR branches have negative effects on IRE1 activity that include PERK [79] and ATF6 [88]. Nevertheless, the implications of this during ER stress are that XBP1 controls its own levels and cell fate by limiting IRE1 activity.

Furthermore, numerous reports proposed that PUMA as an important and PERK-related contributor to UPR-related cell death [89–91], since it can inhibit all prosurvival Bcl-2 family members and activate the intrinsic pathway of apoptosis [70, 71]. Hence, it is plausible that by preventing *BBC3* (*PUMA*) accumulation, XBP1 modulates the extent of intrinsic apoptotic signaling and thus contributes to the adaptive UPR response. Although our data clearly show that *XBP1s* induction leads to a reduction of PUMA protein levels, and at the same time reduces the extent of apoptotic UPR signaling (Fig. 6), evaluating the exact contribution of XBP1’s role will require further study.

Taken together, our approach not only confirmed previously known XBP1 roles during the UPR [24], but also identified novel targets of this transcription factor that regulate the mechanisms of the UPR cell fate decisions. Having said that, our experimental approaches are limited by the complexity of UPR signaling pathways and all the other factors involved in this process, and therefore, further studies will be necessary to understand the complex relationships between XBP1 and all of its targets. One of the limitations here is that the time (at least 24 h) required to obtain sufficient XBP1 expression prevents the model from reproducing the acute stress response since IRE1 is transiently activated and rapidly downregulated by the PERK arm of the stress response [78].

In summary, the studies presented here have identified that XBP1 can repress expression of two key players involved in UPR, *IRE1* and *PUMA*, and further studies are obviously required to decipher the molecular mechanisms underlying this observation.

The results from three independent experiments are normalized to *GAPDH* and *RPLP0* mRNA levels and expressed as a fold change (FC) over the respective controls. For induction experiments during ER stress, normalization was performed against noninduced and non-ER stress exposed cells, whereas for silencing experiments, the noninduced negative control siRNA-transfected cells that were not exposed to ER stress were used as a control. Significant changes (*P *value* P* < 0.05) are marked in grey. The Supplemental Table 2AB contains all the individual values.

### Supplementary Information

Below is the link to the electronic supplementary material.

Supplementary file1 (PDF 549 KB)

Supplementary file2 (PDF 384 KB)

Supplementary file3 (PDF 319 KB)

Supplementary file4 (PDF 250 KB)

Supplementary file5 (XLSX 12 KB)

Supplementary file6 (XLSX 58 KB)

Supplementary file7 (PDF 219 KB)

## Data Availability

Deep sequencing data were deposited in Gene Expression Omnibus (GEO) at accession number: GSE160416.

## Abbreviations

*ACLY*: ATP citrate lyase
ANK2: Ankyrin 2
*ATF3*: Activating transcription factor 3
*ATF6*: Activating transcription factor 6
*BBC3*: *BCL2*-Binding component 3
BiP: Binding immunoglobulin protein (glucose-regulated protein 78, a.k.a. *HSPA5*)
BSA: Bovine serum albumin
*CALR*: Calreticulin
*CDK6*: Cyclin-dependent kinase 6
*CDKN1A*: Cyclin-dependent kinase inhibitor 1A (a.k.a p21)
*CEBPB*: CCAAT-enhancer-binding protein beta (a.k.a *C/EBP*)
*CHAC1*: ChaC glutathione-specific gamma-glutamylcyclotransferase 1
*CHOP*: CCAAT-enhancer-binding protein homologous protein (a.k.a *DDIT3*)
*CLIP2*: Cytoplasmic linker 2
CTRL: Control
*DDIT3*: DNA damage-inducible transcript 3 (a.k.a CHOP)
*DHCR24*: 24-Dehydrocholesterol reductase
DMSO: Dimethyl sulfoxide
*DNAJA1*: DnaJ heat shock protein family (Hsp40) member A1
*DNAJB2*: DnaJ heat shock protein family (Hsp40) member B2
*DNAJB9*: DnaJ heat shock protein family (Hsp40) member B9 (a.k.a *ERdj4*)
*DNAJC3*: DnaJ heat shock protein family (Hsp40) member C3
*DSP*: Desmoplakin
*DUSP6*: Dual-specificity phosphatase 6
ECL: Enhanced chemiluminescence
*EDEM1*: ER degradation-enhancing alpha-mannosidase-like protein 1
*EGR1*: Early growth response 1
eIF2α: Eukaryotic translation initiation factor 2a
ER: Endoplasmic reticulum
ERAD: Endoplasmic reticulum-associated degradation
ERLEC1: ER lectin 1
*ERN1*: Endoplasmic reticulum to nucleus signaling 1 (a.k.a. *IRE1*)
FOXJ2: Forkhead box J2
*GADD34*: Growth arrest and DNA damage-inducible protein (a.k.a *PPP1R15A)*
*GADD45A*: Growth arrest and DNA damage-inducible alpha
*GADD45B*: Growth arrest and DNA damage-inducible beta
*GAPDH*: Glyceraldehyde 3-phosphate dehydrogenase
GEO: Gene expression omnibus
HEK-293 cells: Human embryonic kidney cells
HeLa: Human cervix adenocarcinoma cells
*HSPA1B*: Heat shock protein family A (Hsp70) member 1B
*HSPA5*: Heat shock protein family A (Hsp70) member 5, (a.k.a. *BIP*)
*HSPA6*: Heat shock protein family (Hsp70) member 6
*HSPA8*: Heat shock protein family A (Hsp70) member 8
HUVECs: Human primary endothelial cells
*ICAM1*: Intercellular adhesion molecule 1
*IL1A*: Interleukin 1α
*IL6*: Interleukin 6
*IRE1*: Inositol-requiring protein 1
MAP3K7: Mitogen-Activated Protein Kinase Kinase Kinase 7
MCF-7: Human breast cancer cell line
*MIST1*: Basic helix–loop–helix family member A15
NFKB2: Nuclear factor kappa B subunit 2
NGS: Next-generation sequencing
PBS: Phosphate**-**buffered saline
*PERK*: Protein kinase RNA-like ER kinase
*PUMA*: BCL2-binding component 3 (a.k.a. BBC3)
qPCR: Quantitative polymerase chain reaction
qRT-PCR: Quantitative real-time PCR
RLU: Relative light units
*RCAN1*: Regulator of calcineurin 1
*RPLP0*: Neutral ribosomal phosphoprotein P0
RTCA: Real-time cell analysis
SD: Standard deviations
SEC23B: Coat complex II component
SERCA: Sarco/endoplasmic reticulum Ca2 + ATPase
siRNA: Small interfering RNA
*SNAI1*: Snail family transcriptional repressor 1
Tg: Thapsigargin
Tm: Tunicamycin
UPR: Unfolded protein response
*WARS*: Tryptophanyl-tRNA synthetase
*XBP1*: X-box-binding protein 1
*XBP1s*: Spliced X-box-binding protein 1
*XBP1u*: Unspliced form of XBP1
*ZNF432*: Zinc finger protein 432
