# Supplementary material for: Genome-wide mRNA profiling identifies X-box-binding protein 1 (XBP1) as an IRE1 and PUMA repressor
Source: Cell Mol Life Sci. 2021 Oct 12;78(21-22):7061–80. doi: 10.1007/s00018-021-03952-1 (PMC8558229; doi:10.1007/s00018-021-03952-1)
Supplement: Supplementary file 1 — Supplementary file1 (PDF 549 KB) [file 18_2021_3952_MOESM1_ESM.pdf]

Supplemental Figure S1

A

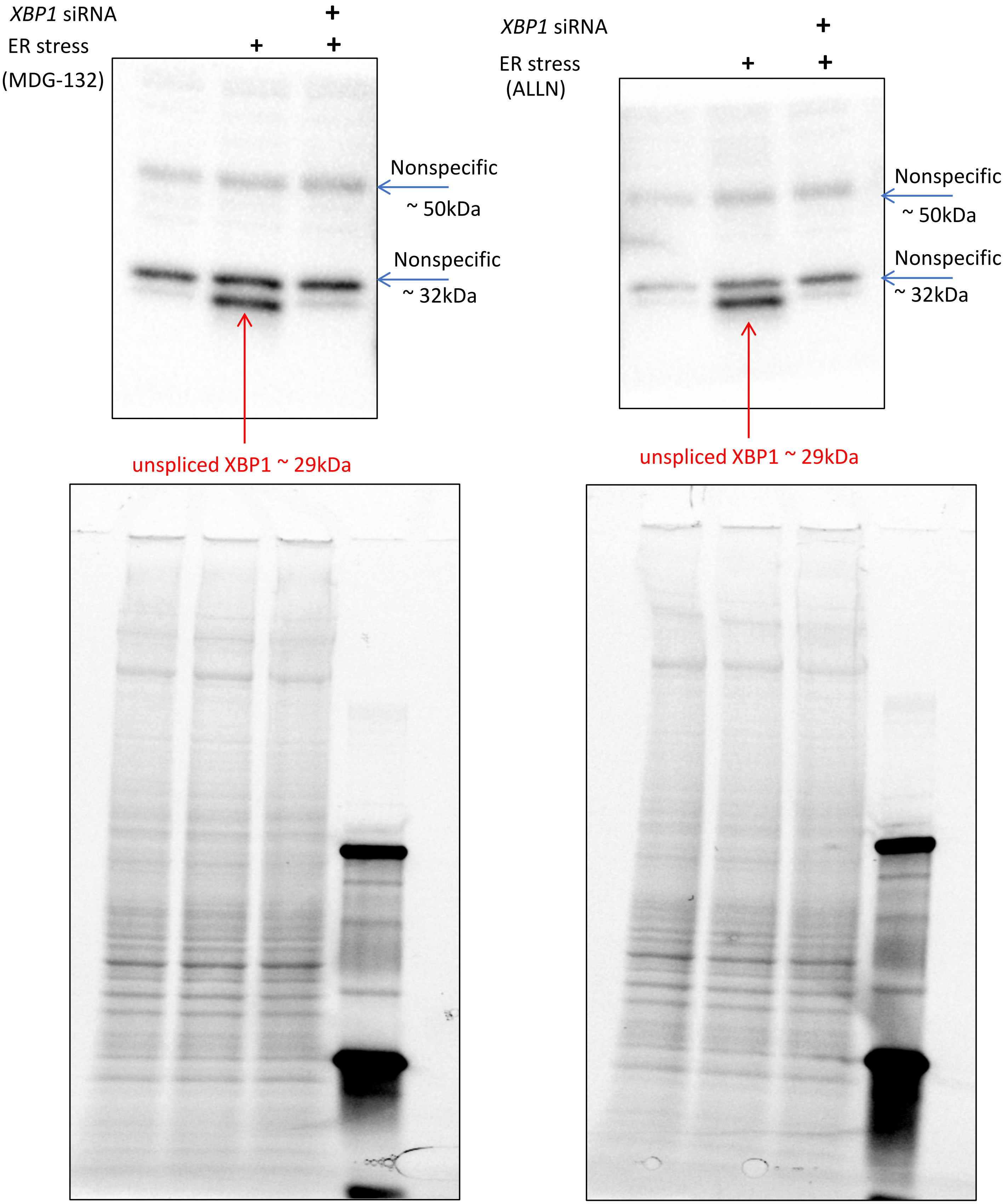

Figure S1A. Validation of antibody against **unspliced** XBP1 (NBP1-77681; Novus Biological USA, diluted at 1:700) with siRNA against XBP1 (Ambion assay id s14915). To induce ER stress and prevent proteasomal degradation of unspliced XBP1, HeLa cells were treated for 6 hours with calpain and proteasome inhibitors MDG-132 and ALLN (Sigma) at 2 μM and 100 μM final concentrations, respectively.

B

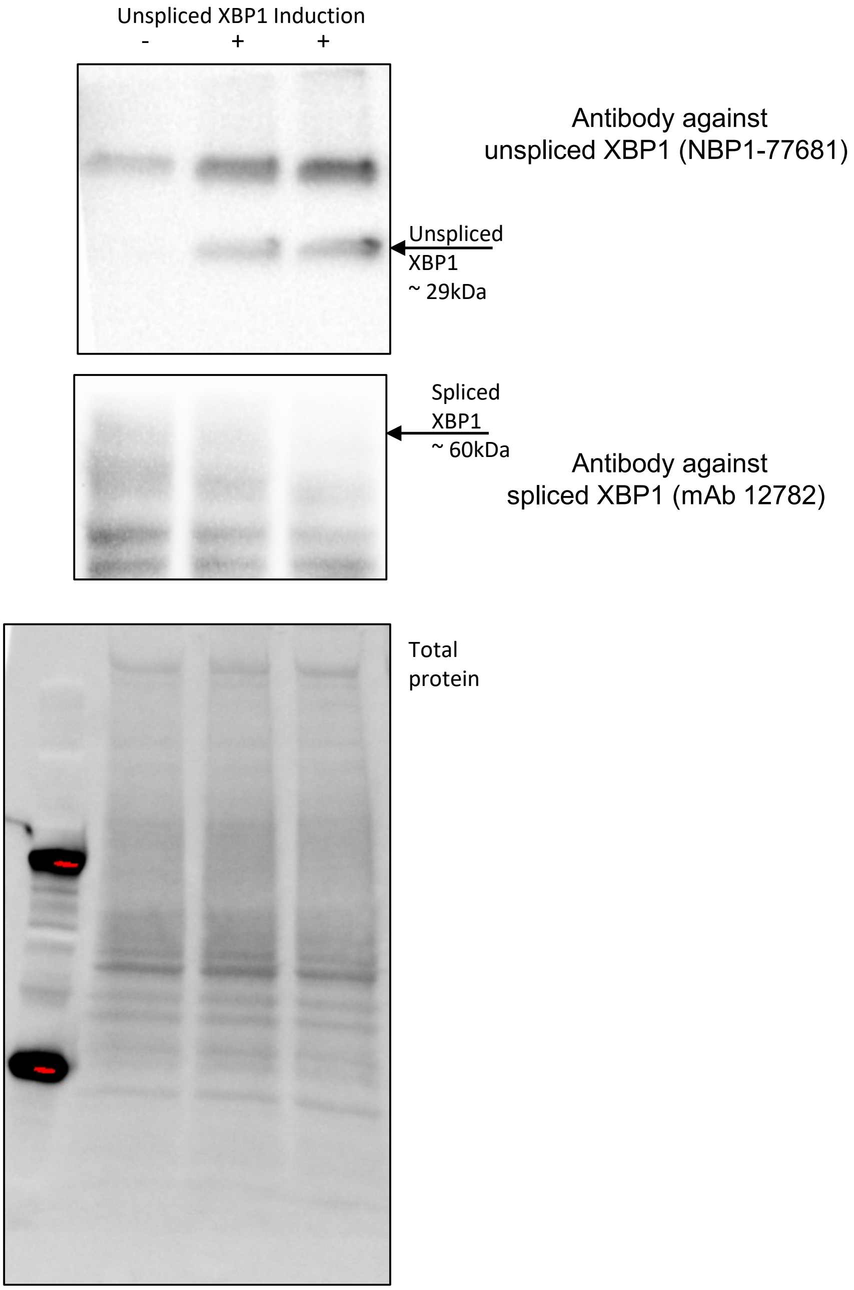

Figure S1B. Upon 24 hours induction, the HeLa-XBP1u cell line accumulates XBP1u but not XBP1s as shown in WB analysis**.**

C

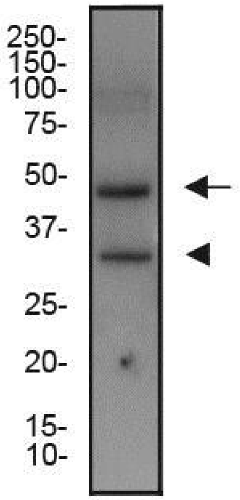

Producer description:
Western Blot: XBP1 Antibody [NBP1-77681] - Total protein from HeLa cells was separated on a 12% gel by SDS-PAGE, transferred to PVDF membrane and blocked in 5% non-fat milk in TBST. The membrane was probed with 1.0 ug/ml anti-XBP1 in block buffer and detected with an anti-rabbit HRP secondary antibody using chemiluminescence. Arrow delineates XBP1s and arrowhead XBP1u.
https://www.novusbio.com/products/xbp1-antibody_nbp1-77681#protocols-faqs

**Immunogen**	A genomic peptide made to an internal region of the human XBP1 protein (within residues 100-250). [Swiss-Prot P17861]
**Localization**	Nucleus
**Specificity**	This antibody is specific for both XBP1s and XBP1u.

Figure S1C. Novus Biologicals description for XBP1 Antibody [NBP1-77681].
