# Supplementary material for: Genome-wide mRNA profiling identifies X-box-binding protein 1 (XBP1) as an IRE1 and PUMA repressor
Source: Cell Mol Life Sci. 2021 Oct 12;78(21-22):7061–80. doi: 10.1007/s00018-021-03952-1 (PMC8558229; doi:10.1007/s00018-021-03952-1)
Supplement: Supplementary file 2 — Supplementary file2 (PDF 384 KB) [file 18_2021_3952_MOESM2_ESM.pdf]

Supplemetal Figure S2

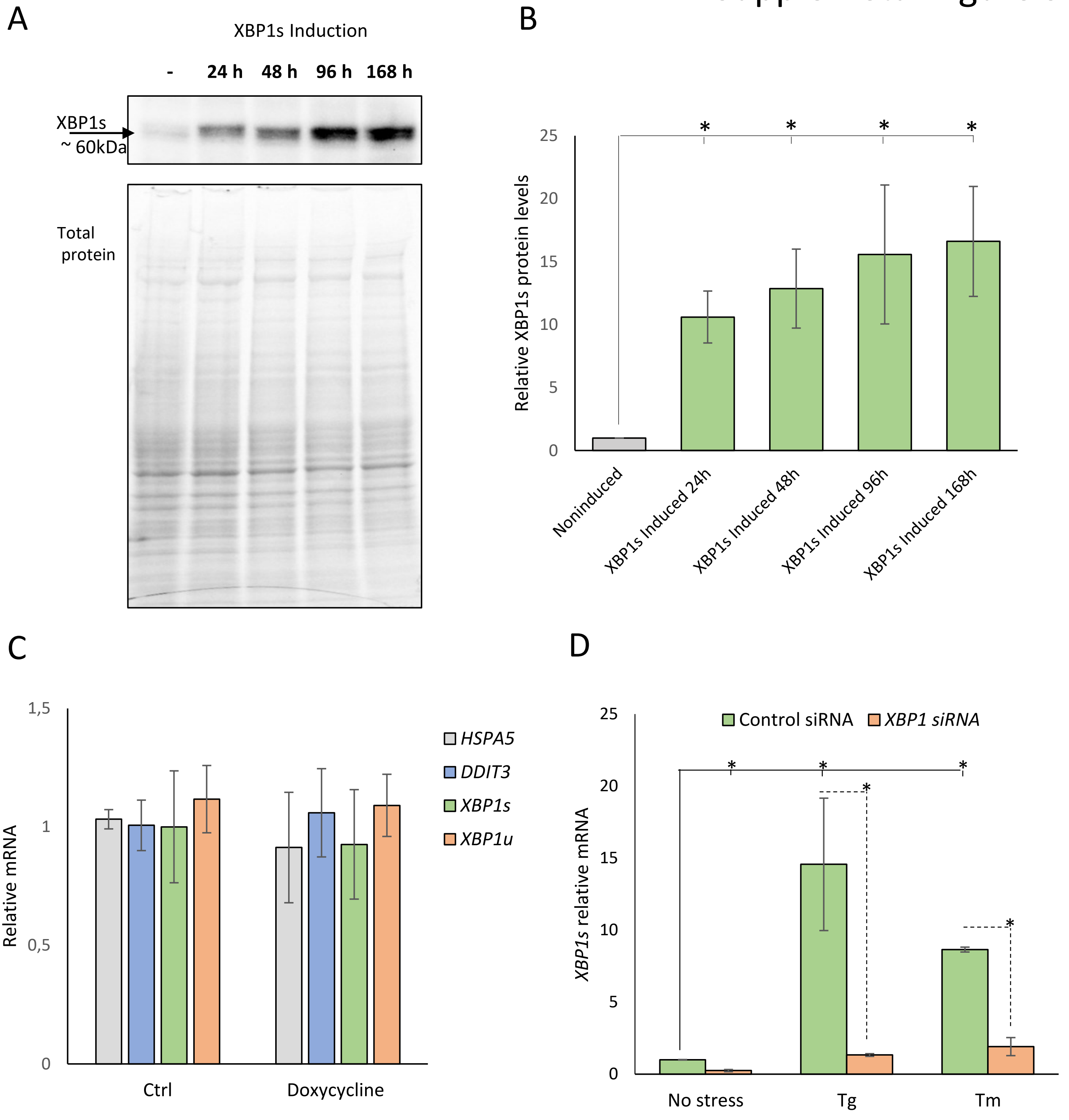

Supplemental Figure 2. Changes in XBP1 protein levels following induction up to one week (168 hours, media supplemented with doxycycline was exchanged every 48 h) were evaluated by Western blot. (**A**) normalized to total protein levels and the noninduced control (**B**). Error bars represent standard deviations. Significant changes (*P-value P*< 0.05) are marked with an asterisk. **Doxycycline used for 24 h XBP1 induction does not induce expression of ER stress markers (*HSPA5, DDIT3*), *XBP1s* or *XBP1u* mRNA levels (C).** The siRNA against *XBP1* allowed specific silencing of XBP1s during ER stress conditions (Tg and Tm induced) (**D**). The results from 3 independent experiments (n=9) are plotted normalized to *GAPDH* and *RPLP0* mRNA levels and expressed as a fold-change over the noninduced cells. Error bars represent standard deviations. Significant changes (*P-value P*< 0.05) are marked with an asterisk.
