# Supplementary material for: Genome-wide mRNA profiling identifies X-box-binding protein 1 (XBP1) as an IRE1 and PUMA repressor
Source: Cell Mol Life Sci. 2021 Oct 12;78(21-22):7061–80. doi: 10.1007/s00018-021-03952-1 (PMC8558229; doi:10.1007/s00018-021-03952-1)
Supplement: Supplementary file 3 — Supplementary file3 (PDF 319 KB) [file 18_2021_3952_MOESM3_ESM.pdf]

Supplemetal Figure S3

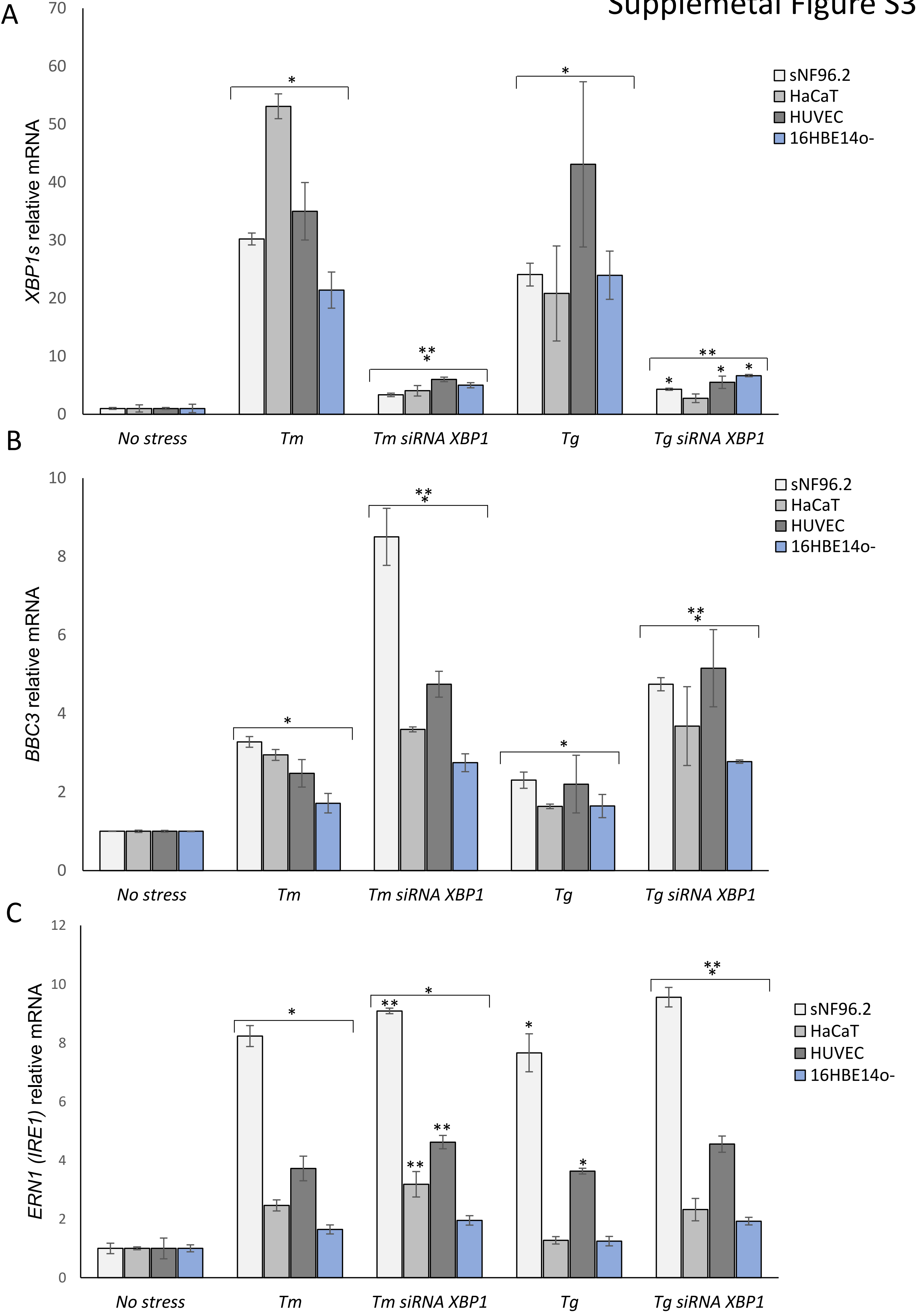

Supplemental Figure S3. Consequences of *XBP1s* silencing (**A**) mRNA levels of *BBC3* **(B)** and *ERN1* **(C),** in different human cell lines that were ER stress exposed for 16 hours (0.5 µg Tm and 2.5nM Tg). Human Schwann cells (SNF96.2) are depicted with light grey, human epidermal keratinocytes (HaCaT) are depicted with grey, 10 donors pooled human umbilical vein endothelial cells (HUVEC) are depicted with dark grey, whereas human bronchial epithelial cells (16HBE14o-) are marked with blue. The results from 3 independent experiments (n=9) are plotted normalized to *GAPDH* and *RPLP0* mRNA levels and expressed as a fold-change over the noninduced cells. Error bars represent standard deviations. Significant changes (*P-value* P < 0.05) are marked with an asterisk (*) versus no stress control and double asterisk (**) versus negative siRNA control in respectful treatments.
