# Supplementary material for: Genome-wide mRNA profiling identifies X-box-binding protein 1 (XBP1) as an IRE1 and PUMA repressor
Source: Cell Mol Life Sci. 2021 Oct 12;78(21-22):7061–80. doi: 10.1007/s00018-021-03952-1 (PMC8558229; doi:10.1007/s00018-021-03952-1)
Supplement: Supplementary file 4 — Supplementary file4 (PDF 250 KB) [file 18_2021_3952_MOESM4_ESM.pdf]

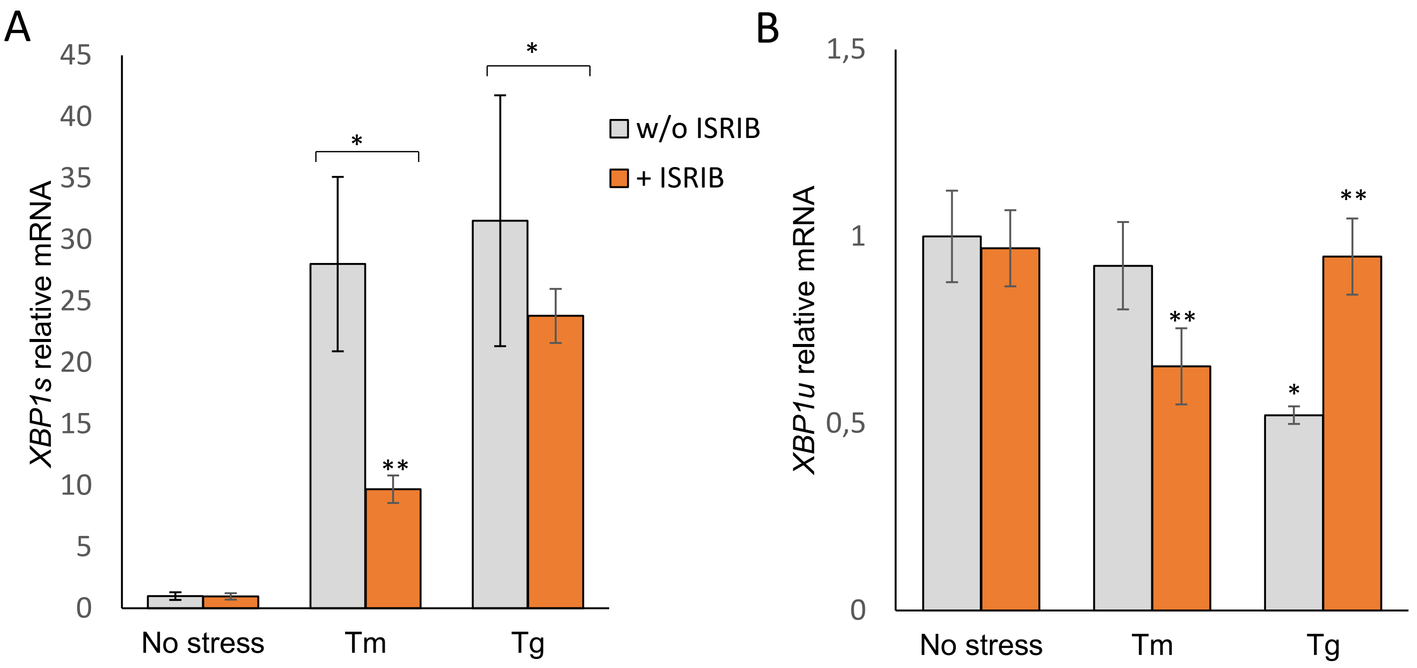

Supplemental Figure S4. Consequences of inhibiting PERK arm of UPR for mRNA levels of *XBP1s* (**A**) and *XBP1u* **(B),** in HeLa cell lines that were ER stress exposed for 6 hours (0.5 µg Tm and 2.5 nM Tg). To prevent PERK signaling integrated stress response inhibitor (ISRIB, Sigma) was used at 1µM concentration. The results from 3 independent experiments (n=9) are plotted normalized to *GAPDH* and *RPLP0* mRNA levels and expressed as a fold-change over the noninduced cells. Error bars represent standard deviations. Significant changes (*P-value* P < 0.05) are marked with an asterisk (*) versus no stress control and double asterisk (**) versus negative siRNA control in respectful treatments.
