# Supplementary material for: Genome-wide mRNA profiling identifies X-box-binding protein 1 (XBP1) as an IRE1 and PUMA repressor
Source: Cell Mol Life Sci. 2021 Oct 12;78(21-22):7061–80. doi: 10.1007/s00018-021-03952-1 (PMC8558229; doi:10.1007/s00018-021-03952-1)
Supplement: Supplementary file 7 — Supplementary file7 (PDF 219 KB) [file 18_2021_3952_MOESM7_ESM.pdf]

Table S3

Gene_id	Gene name	Trancription Factors Motifs Count						
		ATF3	ATF4	ATF5	ATF6	XBP1	CHOP	JUN
43511	*ACLY*	0	2	2	0	1	2	2
34756	*ATF3*	4	13	5	9	6	0	32
2136	*BBC3*	1	6	1	3	4	1	11
730	*CALR*	4	12	2	7	14	0	10
56595	*CDK6*	0	2	2	3	5	0	0
46049	*CDKN1A*	2	6	1	6	10	1	32
53401	*CHAC1*	1	6	0	1	1	3	1
56332	*CLIP2*	0	8	4	1	12	5	25
31831	*DHCR24*	0	0	2	0	2	0	15
7329	*DNAJA1*	4	7	1	2	4	1	6
20511	*DNAJB2*	1	2	2	2	4	0	11
56996	*DNAJB9*	0	2	0	5	3	0	1
45108	*DSP*	0	1	0	4	2	0	0
16419	*DUSP5*	1	11	7	17	12	3	23
22962	*DUSP6*	0	1	0	2	4	0	1
35560	*EDEM1*	1	1	2	7	7	1	12
12699	*EGR1*	2	4	2	4	1	1	1
17688	*ERLEC1*	0	3	1	3	6	3	4
44330	*ERN1*	0	1	1	4	4	2	0
21218	*FOXJ2*	1	4	0	0	4	0	1
32034	*GADD45A*	0	8	1	5	2	2	26
155	*GADD45B*	0	10	0	4	2	4	35
45824	*HSPA1B*	1	4	2	6	13	7	9
8793	*HSPA5*	4	8	3	6	11	2	21
33781	*HSPA6*	0	2	0	0	5	0	8
51073	*HSPA8*	0	0	0	2	1	0	0
547	*ICAM1*	0	1	0	2	4	0	1
18890	*IL1A*	1	3	0	1	3	0	7
55328	*IL6*	2	6	5	2	4	2	14
14031	*MAP3K7CL*	0	0	2	5	4	0	0
16306	*NFKB2*	4	6	2	4	11	0	17
14190	*RCAN1*	0	2	0	6	6	0	6
3351	*SEC23B*	1	5	2	0	3	1	0
4085	*SNAI1*	1	1	2	1	5	1	0
3011	*TRIB3*	0	4	1	2	5	1	12
25822	*WARS*	0	6	3	4	3	7	15
2219	*PPP1R15A (GADD34)*	6	11	8	12	14	9	50
2472	*ZNF432*	0	0	0	3	3	0	0
